# Supplementary material for: Coral reefs benefit from reduced land–sea impacts under ocean warming
Source: Nature. 2023 Aug 9;621(7979):536–42. doi: 10.1038/s41586-023-06394-w (PMC10511326; doi:10.1038/s41586-023-06394-w)
Supplement: Supplementary file 1 — Supplementary Information [file 41586_2023_6394_MOESM1_ESM.pdf]

nature portfolio

https://doi.org/10.1038/s41586-023-06394-w

Supplementary information

Coral reefs benefit from reduced land–sea impacts under ocean warming

In the format provided by the authors and unedited

nature portfolio

https://doi.org/10.1038/s41586-023-06394-w

Supplementary information

Coral reefs benefit from reduced land–sea impacts under ocean warming

In the format provided by the authors and unedited

Supplementary Information for

Coral reefs benefit from reduced land-sea impacts under ocean warming

Jamison M. Gove*, Gareth J. Williams*, Joey Lecky, Eric Brown, Eric Conklin, Chelsie W. W. Counsell, Gerald Davis, Mary K. Donovan, Kim Falinski, Lindsey Kramer, Kelly Kozar, Ning Li, Jeffrey A. Maynard, Amanda McCutcheon, Sheila A. McKenna, Brian J. Neilson, Aryan Safaie, Christopher Teague, Robert Whittier, and Gregory P. Asner

*Corresponding authors. Email: jamison.gove@noaa.gov, g.j.williams@bangor.ac.uk

Table of Contents

Supplementary Methods 2

Coral Reef Fish and Benthic Surveys 2

Statistical Analyses 4

Geospatial Analyses 18

Local land-sea human impacts and environmental factors 19

References 39

Supplementary Methods

Coral Reef Fish and Benthic Surveys

Coral reef fish and benthic surveys were conducted between 2003 and 2019 by three separate monitoring programs. The protocols for each program are as follows.

State of Hawaiʻi Division of Aquatic Resources – Surveys of fish biomass and benthic cover were conducted at a total of 23 sites across West Hawaiʻi beginning in 2003. One site was added in 2005 along with another in 2007. At each site, four separate 25 m x 4 m belt transects were demarcated via stainless steel eye-bolts affixed to the substrate to ensure that the same swath of reef was resampled during each subsequent survey. The four transects were arrayed in the alongshore direction in an "H" pattern with two deeper and two shallower transects along the reef shelf. Each transect was separated from all others by approximately 10 m.

Fish surveys were conducted at all sites four times per year, nominally during the months of May, July, September, and November. During each visit, two teams of two divers surveyed all four transects in a single dive (two transects per team). Before beginning the survey, divers extended nylon lines along the full 25 m lengths of the transects. Surveyors then completed one 'high swim', traversing the full transect length while identifying, counting, and sizing all highly mobile species within the transect area. Divers then turned around and completed a 'low swim', identifying, enumerating, and sizing all remaining species, which tend to be those that are more cryptic or benthic-associated. Fishes were sized according to a binned scheme wherein fishes with total length (TL) ≤25 cm were assigned a letter corresponding to a 5 cm bin (A = 0.1-5 cm, B = 5.1-10 cm, …, E = 20.1-25 cm). TL for fishes > 25cm were rounded to the nearest 5 cm starting at 30 cm. Along each pass, divers swam pairwise along the transect such that each was responsible for a separate 25 m x 2 m belt. Data from individual divers were summed together to yield estimates for a full 25 m x 4 m belt.

Benthic surveys were conducted in 2003, 2007, 2011, 2014, 2016, and 2017. At each site, the transects were set up in the same manner as for fish surveys. A benthic camera operator then collected photoquadrats using an Olympus camera with a 0.75 m monopod attached to keep a consistent elevation above the benthos. Photoquadrats were collected at every m mark along all four transects starting at 0 m. Thus, each transect had a total of 26 images for a total of 104 images per site. Photos were analysed post-dive via either Photogrid (2003) or Coral Point Count with Excel extensions (CPCe; 2007-2017). For the photo analysis process, 20 points were randomly overlaid on each image and the benthic cover was annotated for each point to the lowest possible taxonomic rank.

National Park Service – A split panel design was used with 30, 25 m x 5 m belt transects in the Kaloko-Honokōhau National Park. Transect locations were randomly selected using ArcGIS® and included all fore-reef slope, hard bottom communities between 10 and 20 m depths within the park's legislated boundaries. This depth range was selected for ecological and dive safety reasons. Fifteen fixed (permanent) transects were randomly selected at the onset of the monitoring program in 2007 and marked with stainless steel pins for relocation purposes. These sites were subsequently re-sampled each year. The remaining 15 transect locations were randomly selected each year of monitoring and sampled only once (temporary). Sampling was

typically conducted from September - November. Only the fixed sites are used in this analysis for the years 2014, 2016, 2017, 2018, and 2019. All fish and benthic data collection were co-located and co-visited at each of the sites using the same SCUBA dive team.

Fish surveys were conducted at each permanently marked site with a 25 m transect positioned typically parallel to shore along a constant depth contour. One fish observer identified, enumerated, and estimated the TL (to the nearest cm) of all fishes encountered along the distance of the transect from the bottom to the surface in the 5 m belt. The location, bearing, survey date, and depth of transects were recorded after each dive. To minimise observer bias, sizing calibration dives were conducted using fish models of known size at the beginning of each field season. Observer crossover training was done using two observers side by side when possible.

Benthic surveys were conducted by a diver that followed the fish observer and photographed the substrate at 1-m intervals along the 25 m transect at a perpendicular height of 0.5 m above the substrate. Post hoc image analysis of the photoquadrats was conducted using Photogrid® software (or other suitable software such as CPCe) with 50 randomly selected points per quadrat image. Per cent cover was tabulated (by lowest possible taxon, preferably species) for coral, macroinvertebrates, and other benthic substrate types (e.g., crustose coralline algae, turf algae, fleshy macroalgae, or sand).

The Nature Conservancy – Coral reef monitoring surveys were designed and conducted to assess reef resilience along West Hawai‘i[1]. A total of twenty monitoring locations were identified and selected in consultation with the State of Hawai‘i Division of Aquatic Resources. Most locations were spaced approximately ~2.5 kilometres apart except for survey locations at Honokōhau and Keauhou. All locations had a shallow (5–7 m) and deep (12–15 m) monitoring site, totalling 40 sites. Surveys predominantly occurred in October 2015, 2016, 2018, and 2019. Because the 2015 surveys were performed during the marine heatwave, coral bleaching was widespread and severe. For our analysis, no distinction was made between bleached and un-bleached corals (all included in estimates of coral cover) in our assessment of pre-disturbance benthic cover. All sites except Honokōhau were marked with permanent stainless-steel eyebolts beginning in 2016. Site photos, field notes, and compass bearings collected in 2015 were used to align installation of transect markers.

Fish surveys (25 m x 5 m belt transects) were collected while slowly deploying parallel 25 m transect lines. Each diver identified, enumerated, and estimated the sizes into 5 cm bins (i.e., 0 - 5 cm, > 5 - 10 cm, > 10 - 15 cm, etc.) all fishes within or passing through a 5 m belt. All fish surveys were conducted by trained divers to reduce surveyor variability. Benthic surveys were conducted along each 25 m fish survey transect by photographing the bottom every m using a Canon Powershot camera or equivalent. The camera was mounted on a 0.8 m PVC monopod to ensure consistent elevation above the benthos. At total of 25 images for each survey site were collected, with each photo covering approximately 0.8 m x 0.6 m of the bottom. A 5 cm scale bar marked in 1-cm increments was included in all photographs. Twenty randomly-selected photographs from each transect were analysed to estimate the per cent cover of coral, algae, and other benthic organisms. As needed, selected photographs were imported into Adobe Photoshop CS5 where their colour, contrast, and tone were auto-balanced to improve photo quality prior to analysis. Photos were analysed using CoralNet[2]. Thirty random points were overlaid on each

photograph, and the benthic component under each point was identified to the lowest possible taxonomic level. All photographs were processed by the same analyst to reduce potential observer variability. Once completed, the raw point data from each photograph was combined to calculate the per cent cover of each benthic component for the survey site.

Statistical Analyses

Temporal change – Change over time in local land-sea human impacts and environmental factors for each 100 m section along the 10m isobath (Fig. 1c) were calculated based on the mean difference between the first five years (2000–2004) and most recent five years (2015–2019) in the time series. The 5-year time window was used to account for year-to-year variability in episodic events that are common for drivers such as waves, rainfall, and sediment. The distributions, change over time, and variability for most all drivers are shown in Fig. S1. Fish biomass metrics and phytoplankton biomass data are omitted from Fig. S1 as those data were unavailable at annual time steps. See Extended Data Table 1 for detailed information on local land-sea human impacts and environmental factors, including data collection methods, data sources and ancillary data sets, and specific tools or software utilised.

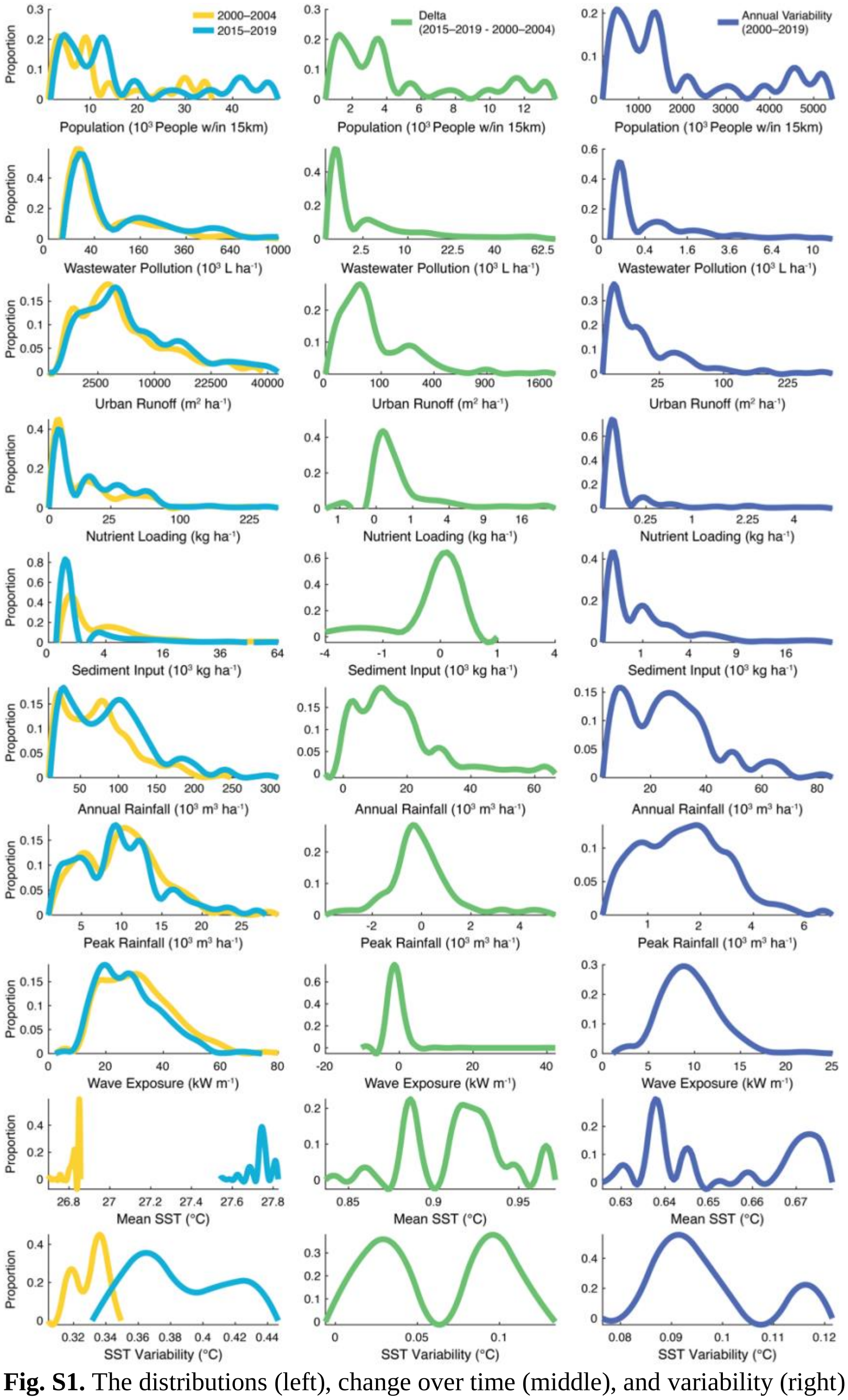

Fig. S1. The distributions (left), change over time (middle), and variability (right) for land-sea human impacts and environmental factors included in our analyses. The distributions in mean values were calculated from the mean of the first five years (2000 – 2004) and the most recent five years (2015 – 2019). Change over time ('delta') represents the most recent five years minus the first five years. Variability was calculated as the standard deviation in annual data from 2000 – 2019. Data shown were geographically constrained to within the northern and southern latitudinal extent of our reef surveys (Fig. 1b). Metrics of fish biomass and phytoplankton biomass are not shown as these data are unavailable at the same temporal or spatial resolution (see Methods). See Extended Data Table 1 for summary information on the local land-sea human drivers and environmental factors included in our analyses. See Supplementary Methods for detailed information on calculating each driver, including data collection methods, data sources and ancillary data sets, and specific tools or software utilised.

Coral reef trajectories pre-disturbance – We assessed the change in coral cover at 23 reefs from 2003 to 2014 (Fig. 2). A reef was considered to have a positive trajectory or negative trajectory if coral cover from the 2003 survey to the 2014 survey increased or decreased by greater than 3%, respectively (Fig. 2b). This cut off was based on the range in mean coral cover among all 23 reefs across the 12-year period (min = 34.1%; max = 36.9%; range = 2.8%).

We then quantified local human impacts and environmental factors at each reef as follows:

- Human population, wastewater pollution, nutrient loading, urban runoff, annual rainfall, peak rainfall, SST mean, SST variability: mean of all data from 2000–2014. This time frame spans 3 years prior to and inclusive of the coral reef benthic data.
- Phytoplankton biomass and irradiance: maximum monthly climatology[3].
- Sediment and wave exposure: mean of the top five events from each year spanning 2000–2014.
- Fishing gear restriction values were assigned to each reef surveyed based on the 2003 marine managed area designation (see *Fishing Gear Restrictions* section below).
- Depth was assigned using in-water diver assessed values and did not change over the 2003 – 2014 time period.
- Fish biomass metrics: mean of all surveys for each year from 2003–2014.

The difference in local human impacts and environmental factors between positive and negative trajectory reefs were then calculated as the difference in the mean drop-one jackknife (using *Jackknife* in Matlab v2021a) values for each impact of factor (Fig 2d; *sensu*[4]). Upper and lower bars in Fig. 2d represent the respective maximum and minimum differences in drop-one jackknife values between positive and negative trajectory reefs. Prior to calculating the drop-one jackknife values, we identified and removed outliers that fell outside a threshold of ± 2 standard deviations of the median. We formally tested for a difference in the local conditions of positive versus negative trajectory reefs using a multivariate permutational analysis of variance (PERMANOVA)[5] based on a Euclidean distance similarity matrix, Type III (partial) sums-of-squares, and unrestricted permutations of the normalised data. We used a modified version of the *pseudo-F* test statistic[5] that better accounted for the variation in dispersion among positive and negative trajectory reefs that was apparent within our data. To achieve this, the PERMANOVA test was completed using updated routines in the PERMANOVA+ add-on[6] for Primer v7[7]. We visualised the results in Fig. 2c using a constrained analysis of principal coordinates (CAP)[8] to capture the multidimensional and correlated nature of the data (Fig. S2), and calculated the cross-validation allocation success (a measure of group distinctness) from the leave-one-out procedure of the CAP analysis. Allocation success was considered indicative of a more distinct set of human-environmental attributes than expected by chance alone when values exceeded 50%. This threshold came from the possibility of each individual observation having a 50% chance of being placed into one of the two groups.

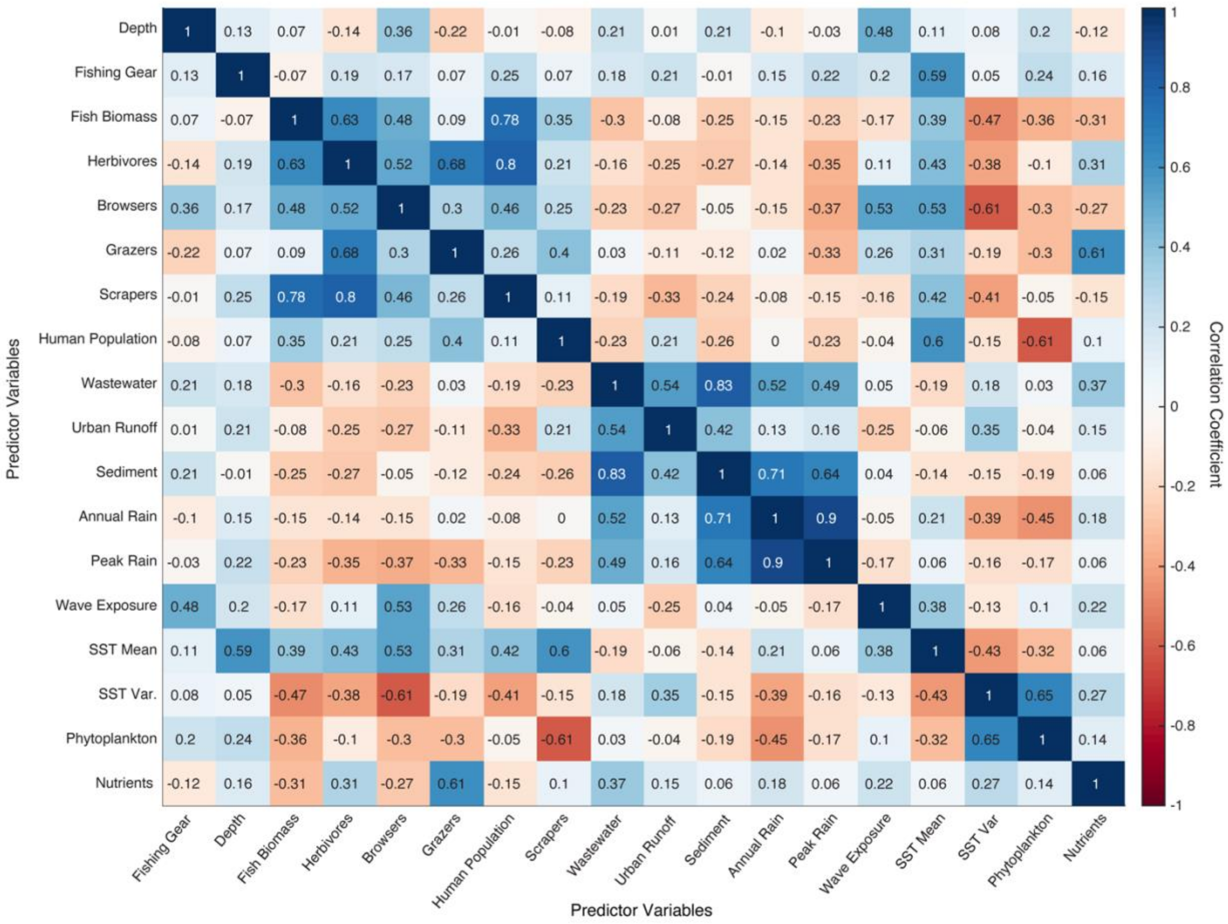

Fig. S2. Pearson correlation (*r*) coefficients for all the local land-sea human impacts and environmental factors used in our canonical analysis of principal coordinates (CAP) analysis to quantify and visualise the relative distinctness in local land-sea human impacts and environmental factors between positive versus negative trajectory reefs over the 12 years pre-disturbance. See Extended Data Table 1 for summary information on local land-sea human impacts and environmental factors included in our analyses. See Supplementary Methods for detailed information on calculating each impact or factor, including data collection methods, data sources and ancillary data sets, and specific tools or software utilised.

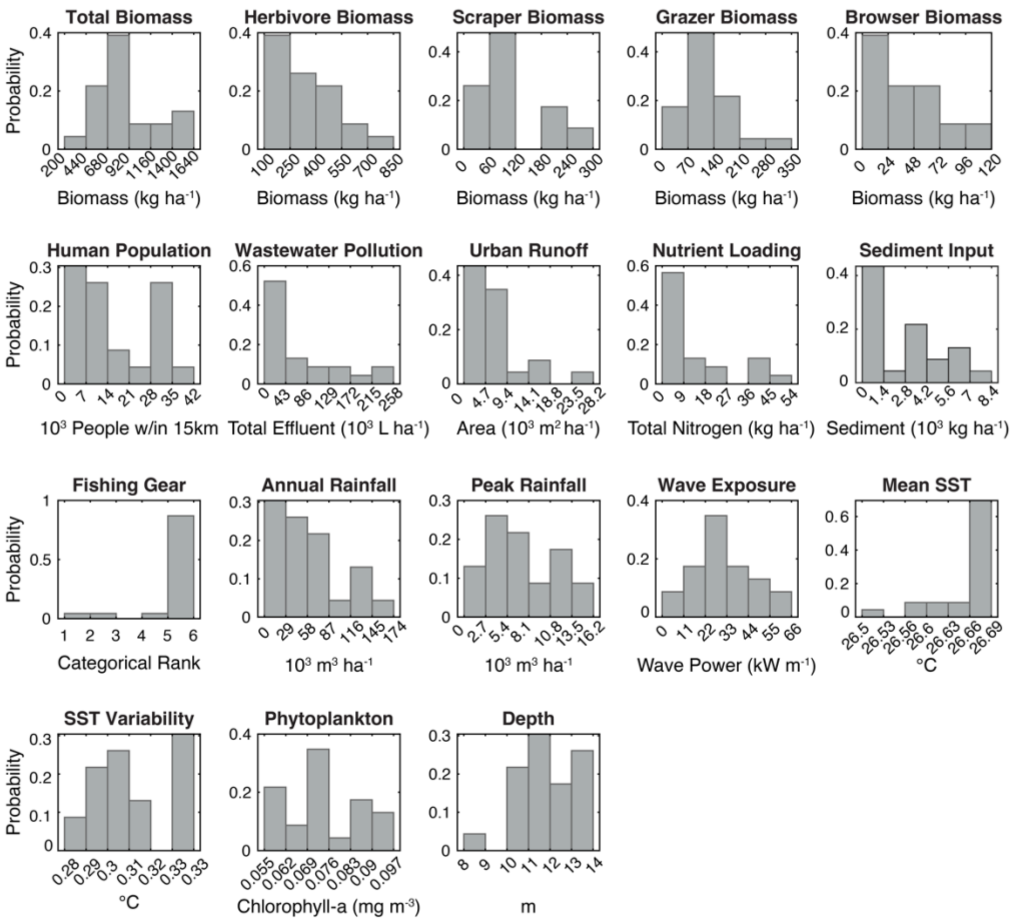

Fig. S3. The underlying data distributions in land-sea human impacts and environmental factors shown in Fig. 2d and Extended Data Fig. 3. See Extended Data Table 1 for summary information on the factors included in our analyses. See Supplementary Methods for detailed information on calculating each factor, including data collection methods, data sources and ancillary data sets, and specific tools or software utilised.

Coral response to the marine heatwave – Our goal was to assess the local land-sea human impacts and environmental factors that best explained changes in coral cover as a consequence of the 2015 marine heatwave. Any potential to observe change, however, could be influenced by variations in starting condition. Reefs with higher initial cover (like those on positive coral cover trajectories pre-disturbance, Fig. 2b) had greater scope for loss, and vice versa[9] (Extended Data Fig. 5). To account for this and ensure greater comparability across reefs (Fig. S4), we calculated coral cover change following[10] as:

$$\%\text{difference}\Delta = [(A_{a,i} - A_{b,i})/A_{b,i}] \times 100$$

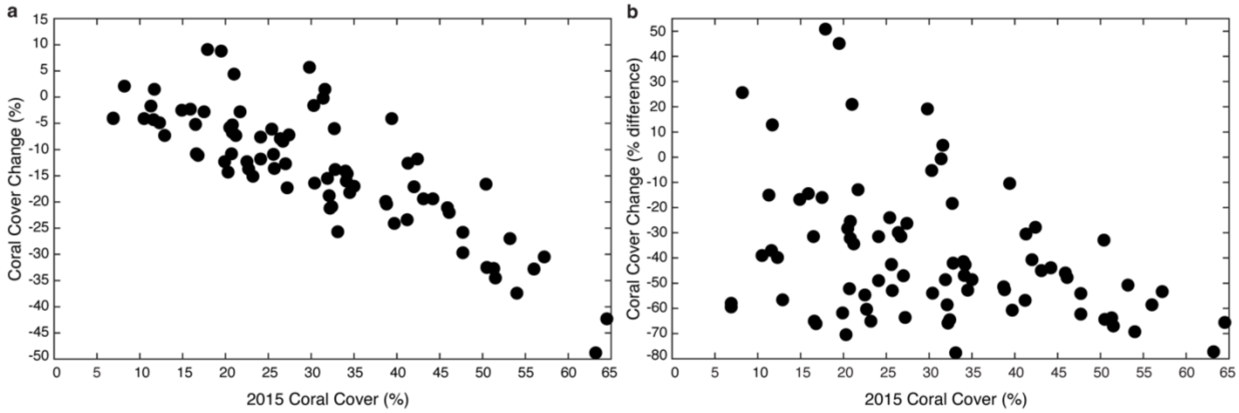

Fig. S4. a, Relationship between coral cover just prior to the onset of the marine heatwave in 2015 (x-axis) with coral cover change between 2015 and 2016 (y-axis) among reefs surveyed (n = 80; see Fig. 1b in main manuscript for reef locations). **b**, same as in **a** but with the relationship between coral cover in 2015 and the per cent difference in coral cover change between 2015 and 2016. The linear relationship in **a** is much reduced as a result of accounting for starting condition when calculating coral cover change in **b**.

We then calculated the following predictors based on current literature and our hypotheses of the principal factors that drive changes in coral cover owing to severe heat stress (Extended Data Table 1):

- Human population, wastewater pollution, nutrient loading, urban runoff, annual rainfall, peak rainfall, wave exposure: mean of all data from 2012–2016. This time frame spans 3 years prior to the marine heatwave through 1 year following, which is inclusive of the 2016 benthic surveys.
- Sediment: mean of the top 3 events from 2006–2016.
- SST mean and SST variability: mean from 2000–2014.
- Degree Heating Weeks (DHW): maximum value for 2015.
- Phytoplankton biomass and irradiance: mean from June – November 2015, representing the time inclusive of anomalous ocean temperatures during the marine heatwave.
- Fishing gear restriction values were assigned to each reef surveyed based on the 2015 marine managed area designation (see *Fishing Gear Restrictions* section below).
- Depth was assigned using in-water diver assessed values and did not change over the 2014–2016 time period.
- Fish biomass metrics: mean of fish data that were coupled with benthic surveys: 2014 (n = 40) or 2015 (n = 40) and 2016 (n = 80).

Prior to model fitting, we identified the presence of outliers in our predictor variables as any point that fell outside a threshold of ± 2 standard deviations of the median. We then applied an additional step to retain any point above this threshold that was within 25% of the maximum predictor value below the threshold. This ensured that no data points were unnecessarily discarded from our formal model-fitting process because of applying an arbitrary threshold cut-off for data inclusion. The following predictors were then square-root transformed to down-weight the influence of values at the extreme ends of their distributions: all fish biomass metrics, wastewater pollution, urban runoff, nutrient loading, phytoplankton biomass, and peak rainfall. A fourth-root transformation was applied to sediment. To reduce model overfitting, Pearson's

correlation coefficients were calculated among all predictors (Fig. S5), removing one of each pair of highly correlated (*r* >0.7) predictors. This resulted in the following predictors being removed from the model fitting process:

- The mean in sea surface temperature (SST Mean) and SST variability owing to their strong correlation (*r* = 0.83 and -0.82, respectively) with Degree Heating Weeks (DWH).
- Annual rainfall owing to the strong positive correlation (*r* = 0.91) with peak rainfall
- Irradiance owing to the positive correlation (*r* = 0.83) with phytoplankton biomass.
- Herbivore biomass owing to the strong positive correlation with grazer and scraper biomass (*r* = 0.76 and 0.74, respectively).

To further strive for model parsimony, we a priori excluded human population from the model fitting process as it was a poor indicator of human driven land-to-sea impacts at local scales (see Fig. 1c in main manuscript). We also excluded browser biomass as they represented <10% on average of total herbivore biomass across all reefs prior to, during, and post-disturbance. Browsers also typically feed on larger fleshy macroalgae that are rare in Hawai‘i[11]. In contrast, we include scrapers and grazers given they target small algal turfs which are the dominant fleshy algae across our study region[12,13].

The remaining 13 predictors were used in the model fitting process (Fig. S6): Total fish biomass, grazer biomass, scraper biomass, wastewater pollution, nutrient input, urban runoff, sediment input, peak rainfall, DHW, wave exposure, phytoplankton biomass, fishing gear restrictions, and depth. The decision of which correlated predictors to retain was based on a hypothesis-driven approach, in part whether the given predictor had the potential to directly (e.g., sediment input) rather than indirectly (e.g., annual rainfall driving sediment input) affect heat-driven coral loss.

We fitted generalized additive mixed-effects models (GAMM) using the gamm4[7] package for R v4.0.2 (www.r-project.org) to model coral cover change from 2014/2015 to 2016. We incorporated a random spatial factor to account for the possible influence of a change in an underlying variable along the coastline not quantified in this study. This was done by breaking the coastline up into discrete 10-km sections running north to south. Section size was determined using hierarchical clustering based on pairwise Euclidean distances between reefs and identifying an inflection point in the intra-group variance (*sensu*[14]; Fig. S7). We fitted GAMMs for all possible candidate models (unique combinations of the predictor variables, with a limit of 6 predictors per model to reduce overfitting) using the UGamm wrapper function, in combination with the dredge function in the MuMIn package[15]. Non-linear smoothness in the models was determined using penalized cubic regression splines, with the number of knots (limited to 4 to reduce overfitting) spread evenly throughout each covariate. We used Akaike's Information Criterion with a bias correction for small sample sizes[16] (AICc) for model comparison and all models within ΔAICc ≤ 2 of the top model (ΔAICc = 0) are presented in Extended Data Table 2. To visualize the effect of predictor terms on coral cover change, we averaged the coefficients from the top models (ΔAICc ≤ 2) to generate a predicted dataset and set all other predictor terms to their median value. Finally, we calculated a measure of predictor variable relative importance within each candidate model by calculating the sum of AICc model weights for each predictor, i.e., the sum of model weights across all models containing each predictor. The relative importance values for all 13 predictors used in the GAMM analysis were as follows: sediment

input (0.99), scraper biomass (0.99), total fish biomass (0.90), urban runoff (0.60), phytoplankton biomass (0.38), wastewater pollution (0.28), peak rainfall (0.20), nutrient loading (0.19), grazer biomass (0.16), DHW (0.08), wave power (0.07), depth (0.06), and fishing gear restrictions (0.05).

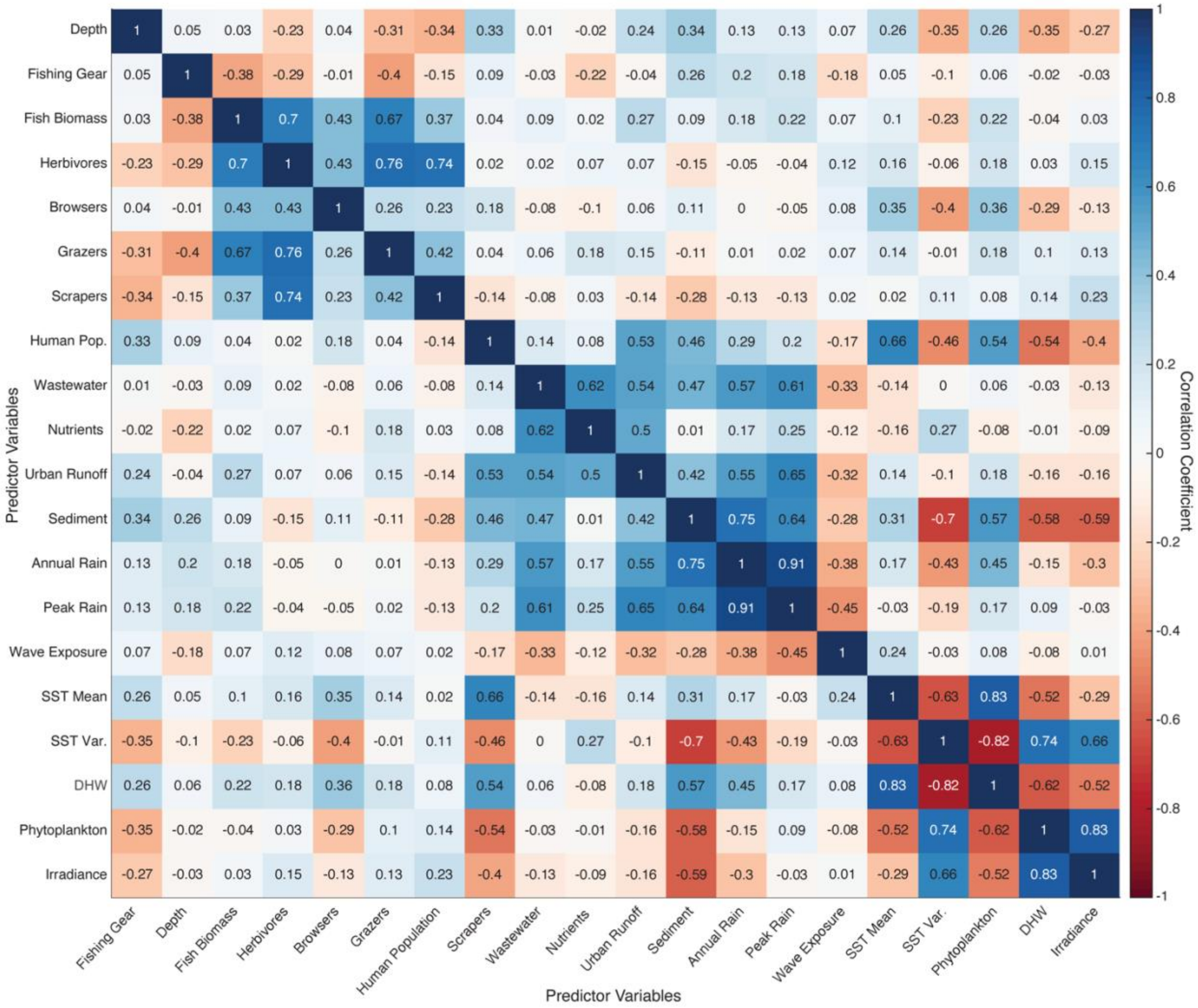

Fig. S5. Pearson correlation (*r*) coefficients for all predictor variables considered for the generalized additive mixed effects modelling (GAMM) relating coral response to the 2015 marine heatwave to local land-sea human impacts and environmental factors. Where $r > 0.7$ for any given pairwise comparison, one of the predictors was removed. We chose the predictor that was considered more interpretable in driving variation in the response variable should it come out as important in the top models. See Extended Data Table 1 for summary information on local land-sea human impacts and environmental factors included in our analyses. See Supplementary Methods for detailed information on calculating each impact or factor, including data collection methods, data sources and ancillary data sets, and specific tools or software utilised.

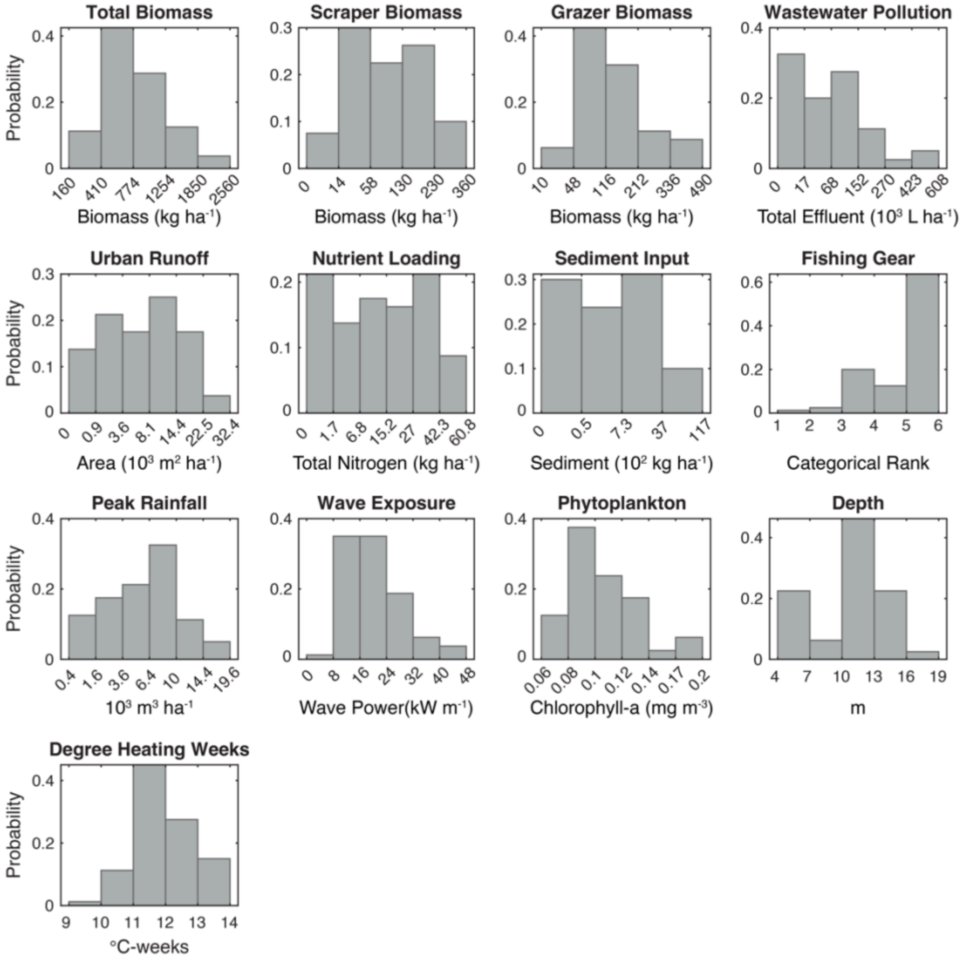

Fig. S6. Predictor variable distributions used in our generalized additive mixed model (GAMM) relating coral response to the 2015 marine heatwave to local land-sea human impacts and environmental factors. See Extended Data Table 1 for summary information on factors included in our analyses. See Supplementary Methods for detailed information on calculating each factor, including data collection methods, data sources and ancillary data sets, and specific tools or software utilised.

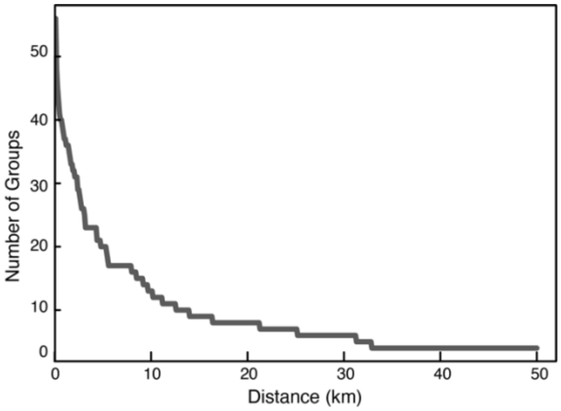

Fig. S7. Number of groupings owing to changes in distance (km) calculated from hierarchical clustering using pairwise Euclidean distances between reefs. The number of unique groups showed a rapid decline up to and over 10 km. We therefore explored section sizes of 10 – 30 km (in 5-km increments) as the random factor in our generalized additive mixed models (GAMM). Regardless of section size, the random factor remained non-significant in all of our models.

Coral reefs four years post-disturbance – Our goal in this analysis was to assess the local land-sea human impacts and environmental factors that best explained variations in the cover of reef-building organisms four years following the 2015 marine heatwave. The cover of reef-building organisms for reefs surveyed in 2019 (n = 55) were parsed into three categories based on the following percentiles: Low, ≤ 25th; Moderate, > 25th & < 75th; High, ≥ 75th. The predictors below were included based on current literature and our hypotheses of the principal factors that drive changes in reef-builder cover across space and time following a major thermal disturbance:

- Wastewater pollution, nutrient loading, urban runoff, annual rainfall, peak rainfall, wave exposure, phytoplankton biomass, and irradiance: mean of all data from 2016–2019.
- Sediment: mean of top 3 events over the 2006–2019 time period
- SST mean & SST variability: mean of all data from 2000–2018. Note that 2019 was excluded owing to the marine heatwave that impacted Hawaiʻi[17], but occurred after our 2019 fish and benthic surveys.
- Fishing gear restriction values were assigned to each reef surveyed based on the 2016 marine managed area designation (see *Fishing Gear Restrictions* section below).
- Depth was assigned using in-water diver assessed values and did not change over the 2016–2019 time period.
- Fish biomass metrics: mean of all surveys from 2016 to 2019.

We used the exact same process as in our GAMM analysis to remove outliers in our predictor variables (see explanation above). We then square-root transformed the following predictors to down-weight the influence of values at the extreme ends of their distributions: total fish biomass, wastewater pollution, sediment input, and nutrient loading. Pearson's correlation coefficients were calculated among all predictors (Fig. S8), removing highly correlated (r > 0.7) predictors. This resulted in the following predictors being removed:

- Phytoplankton biomass owing to the negative correlation (r = -0.71) with urban runoff.
- Annual rainfall owing to the strong positive correlation (r = 0.91) with peak rainfall.
- Herbivore biomass owing to the strong positive correlation with total fish biomass and grazer biomass (r = 0.83 and 0.82, respectively).
- Removal of the mean and variability in SST owing to the negligible range in values (range of 0.1ºC and 0.025ºC among reefs, respectively).

For the reasons outlined in our GAMM analysis and for continuity, we also a priori excluded human population and the biomass of browsers from the model fitting process. The remaining 12 predictors were used in the model fitting process (Fig. S9): Total fish biomass, grazer biomass, scraper biomass, wastewater pollution, nutrient input, urban runoff, peak rainfall, sediment input, wave exposure, irradiance, depth, and fishing gear restrictions. See the '*Local Land-Sea Human Impacts and Environmental Factors*' section below for more details on how each predictor was derived.

We used ordinal logistic regression (OLR) to determine the probability of a given reef having a *Low, Moderate*, or *High* cover of reef-building organisms based on our 12 predictor variables. Logit models are multivariate extensions of generalized linear regression models that provide

parameter estimates via maximum likelihood estimation (MLE) to model the relative log odds of observing a reef-builder cover category or less versus observing the remaining higher categories:

$$ln\left(\frac{P(y_i \le j)}{P(y_i > j)}\right) = C_j + B_1 z_{i1} + \cdots + B_k z_{ik}$$

Here, i indexes each of N observations, with categories y_i, and the left-hand side of the equation is the logit of the probability of a reef-builder category of j or lower, for $j = 1$ (*High*) or 2 (*Moderate*). Reefs with *Low* reef-builder cover contributed to the regression through calculation of the log odds. Each C_j is an MLE-computed model intercept, and each B is the MLE coefficient corresponding to each standardised independent variable z_{ik}, for $k = 1, \ldots, n$, where n is the number of predictors used in a given model. A fundamental component of this model is the assumption of proportional odds, or parallel regression, which implies B_k values are independent of the logit level j. The validity of this parallel regression assumption was ascertained using Brant's Wald test[18], as well as a likelihood ratio test ($\alpha = 0.05$).

All possible candidate models were computed, with the total number of predictors in any given candidate model limited to 4 (to reduce overfitting and to account for the lower response variable replication compared to our GAMM analysis). Models were computed using the multinomial logistic regression function mnrfit in Matlab v2021a. We used AICc for model comparison and all models within ΔAICc ≤ 2 of the top model (ΔAICc = 0) are presented in Extended Data Table 3. We combined this model selection procedure with an interpretation of the covariates to refine our model selection. Simply selecting the model with the smallest AICc (i.e., top-ranking model) could result in a model with an equally sufficient goodness of fit being ignored despite that model potentially containing parameter estimates that are more consistent given the variability in the underlying data. McFadden's pseudo-R^2 was computed for the highest ranked models, and ranged from 0.20 to 0.22. Unlike traditional R^2 values, McFadden's pseudo-R^2 values of 0.2–0.4 represent an excellent fit[19]. Models within $\Delta AIC_c \le 2$ of Model 1 in Extended Data Table 3 demonstrated comparable levels of goodness of fit and parsimony[20,21]. Many of the parameter coefficients within these models were very sensitive to the data and their estimates did not differ significantly from zero ($p < 0.05$). These probabilities were computed using a normal distribution and we compared this outcome with a Student's *t*-test to compute the *p* values, setting the parameter 'EstDisp' equal to 'on' in the mnrfit function. The results remained largely the same using both approaches. Models 1 and 2 contained parameters with coefficient estimates significantly different from zero, namely scraper biomass and wastewater pollution. We therefore examined changes in the probability of a given reef having *Low*, *Moderate*, or *High* reef-builder cover based on variations in these two land-sea predictors (Fig. 4). Probability curves for *High*, *Moderate*, and *Low* were calculated based on changing Scraper Biomass and Wastewater Pollution and holding all other predictors at their mean.

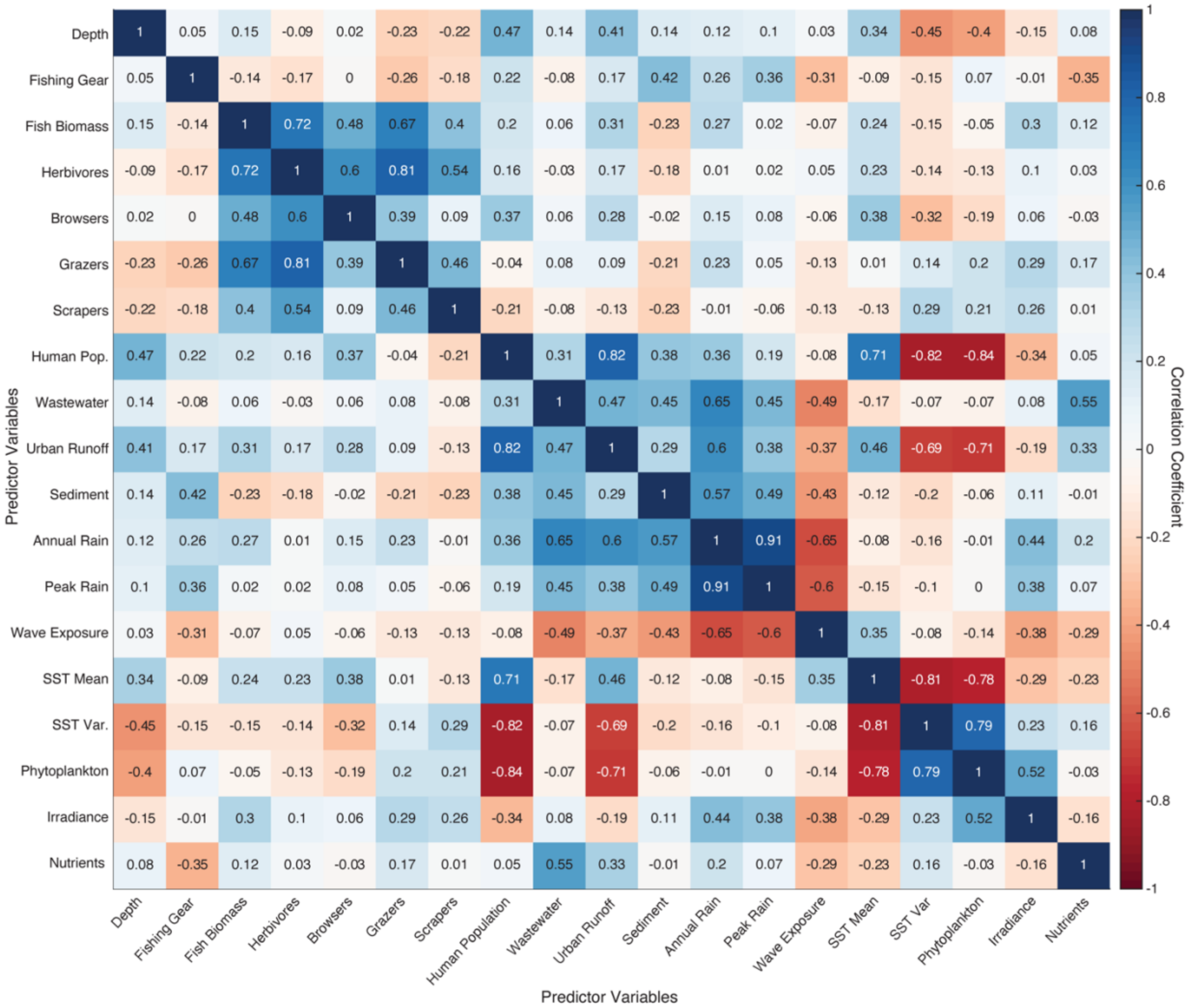

Fig. S8. Pearson correlation (*r*) coefficients for all predictor variables considered for the ordinal logistic regression (OLR) relating the percentage cover of reef-builders (hard coral + crustose coralline algae) four years following the 2015 marine heatwave to local land-sea human impacts and environmental factors. Where $r = > 0.7$ for any given pairwise comparison, one of the predictors was removed. We chose the predictor that was considered more interpretable in driving variation in the response variable should it come out as important in the top models. See Extended Data Table 1 for summary information on local land-sea human impacts and environmental factors included in our analyses. See Supplementary Methods for detailed information on calculating each impact or factor, including data collection methods, data sources and ancillary data sets, and specific tools or software utilised.

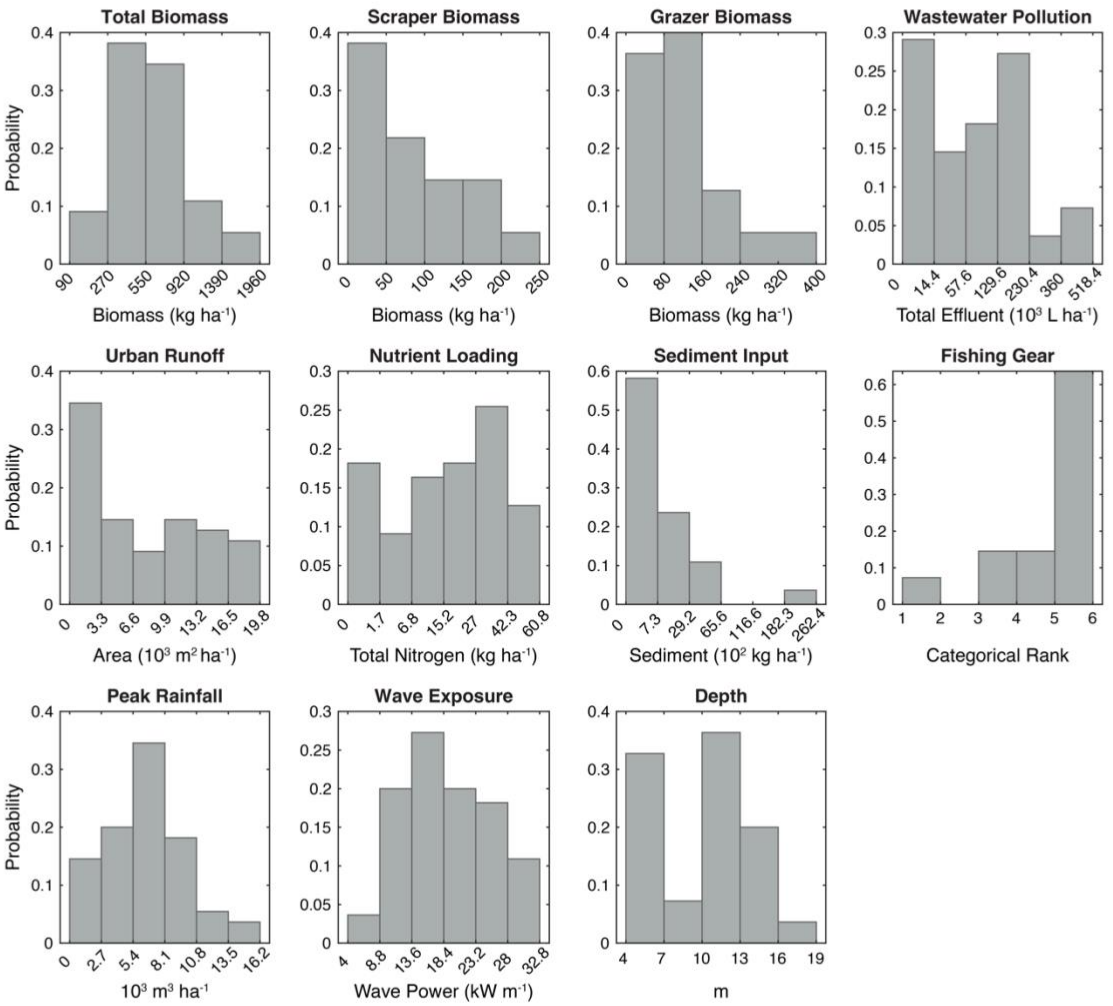

Fig. S9. Predictor variable distributions used in our ordinal logistic regression (OLR) relating the percentage cover of reef-builders (hard coral + crustose coralline algae) four years following the 2015 marine heatwave to local land-sea human impacts and environmental factors. See Extended Data Table 1 for summary information on factors included in our analyses. See Supplementary Methods for detailed information on calculating each factor, including data collection methods, data sources and ancillary data sets, and specific tools or software utilised.

Resource management scenarios – The resource management scenarios presented in Fig. 4b were selected based on the following rationale. We chose 250 kg ha^{-1} as the management target for scraper biomass as this value is close to the biomass of scrapers within Kealakekua Bay, a marine protected area where no fishing has been allowed since 1969. The long-term mean (2003–2019; n = 17) in scraper biomass is 243 kg ha^{-1} while the short-term mean (2016–2019; n = 4) is 302 kg ha^{-1}. Our value is more aligned with the long-term mean to represent a more conservative estimate of scraper biomass on a reef with fisheries protection. In addition, we compared our upper (250 kg ha^{-1}) and lower (30 kg ha^{-1}) scraper biomass values to the distribution of scraper biomass among all reefs (n = 80) in 2019, the most recent time point in which all reefs were surveyed within the same year (Fig. S10). The upper and lower limits represent the 92nd and 36th percentile, respectively. For wastewater pollution, we used our 2019, 100 m grid cell values that fell along the 10m isobath (same as Fig. 1c) but constrained the latitudinal extent to be consistent with the northern- and southern-most locations of the 2019 reef surveys. This approach provided far greater replication and a more representative assessment of

wastewater pollution along the coastline for which to assess our management scenarios. The upper (600,000 L ha^{-1}) and lower (2,500 L h^{-1}) values chosen for wastewater pollution represented the 95th and 36th percentiles of the 2019 distribution, respectively (Fig. S11).

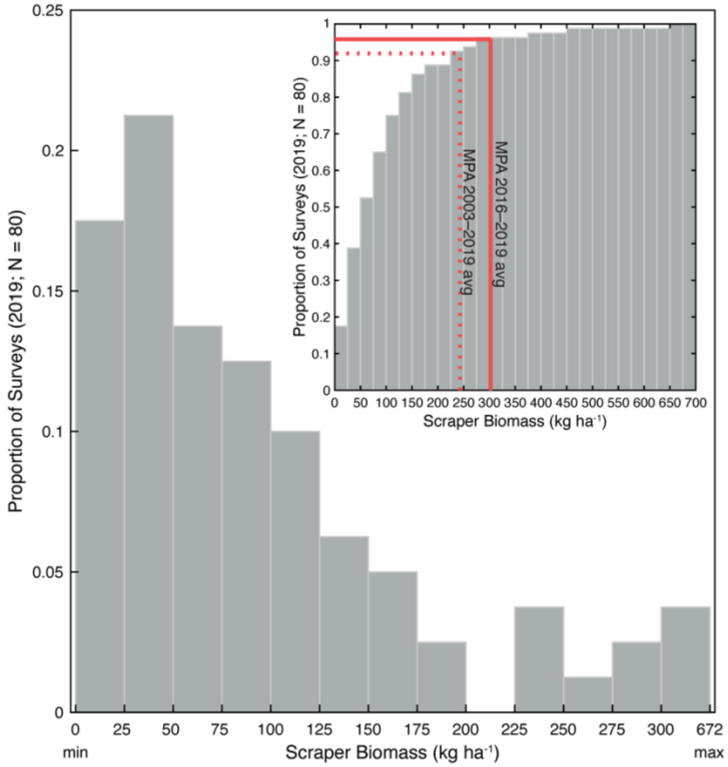

Fig. S10. Distribution of scraper biomass in the most recent year (2019) in which all reefs (n = 80) were surveyed for reef fish. Scraper biomass bars are in 50 kg ha^{-1} intervals except for the last bar that is the sum proportion of all reef surveys between 300–672 kg ha^{-1}, which is the maximum biomass value. Inset figure represents the cumulative density estimate, where the height of each bar is equal to the cumulative relative number of observations in the bar and all previous bars. Scraper biomass from the monitoring site within Kealakekua Bay, a marine protected where no fishing has been allowed since 1969, is shown in red. The vertical dashed and solid red lines represent the long-term average (243 kg ha^{-1}; 2003–2019; n = 17) and recent average (302 kg ha^{-1}; 2016–2019; n = 4) scraper biomass, respectively. The horizontal dashed and solid red lines are the intersection of these respective biomass values along the y-axis, which is the relative proportional or percentile values for the long-term average (0.92) and more recent average (0.93) in scraper biomass.

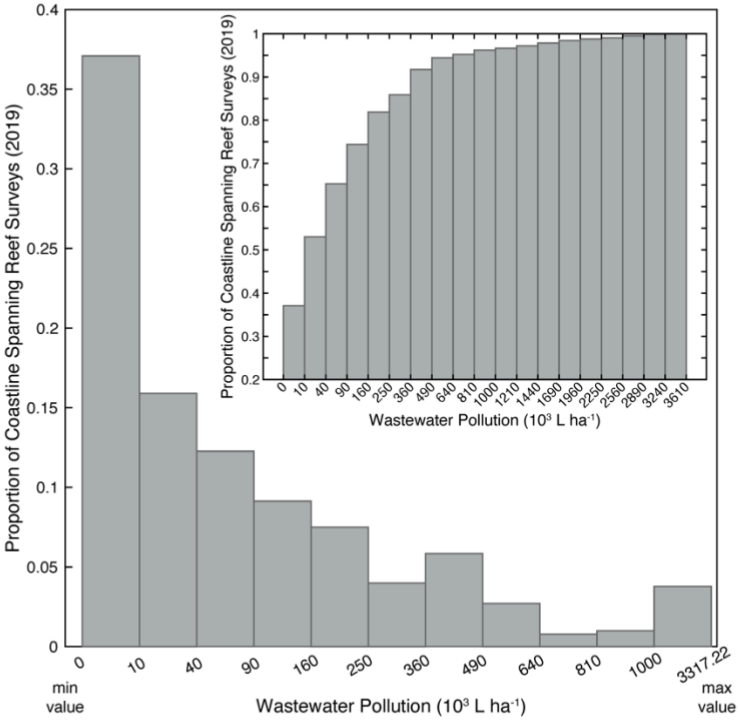

Fig. S11. Distribution of wastewater pollution from our 2019, 100 m grid cell values that fell along the 10 m isobath (same as Fig. 1c) within the northern and southern latitudinal extent of the reef surveys (Fig. 1b). Inset figure represents the cumulative density estimate, where the height of each bar is equal to the cumulative relative number of observations in the bar and all previous bars. The management scenarios for the upper (600,000 L ha^{-1}) and lower (2,500 L h^{-1}) wastewater pollution in our manuscript represented the 95th and 36th percentiles of the 2019 distribution, respectively.

Geospatial Analyses

Unless otherwise specified, all geospatial analyses were conducted in ArcGIS Desktop 10.6 and all tools named below are geoprocessing tools in ArcGIS Desktop.

Raster ocean and land mask – A 100 m resolution data mask differentiating land pixels *versus* ocean pixels was derived from high resolution shoreline data for Hawai‘i Island[22] in coordinate system UTM 4N, NAD1983. Pixels containing any amount of ocean area were given priority during rasterization and designated ocean pixels (i.e., land pixels must contain 100% land area). The ocean portion of the mask extends 5 km offshore – a distance that generously encompasses all shallow coral reef habitat. This data layer was used as the snap raster environment setting to ensure common extent and grid alignment during processing and creation of all final raster layers, as well as to remove land area from intermediate raster datasets when needed. Unless specified otherwise, when vector data (point, line, polygon) was converted to raster it was done at a 100 m resolution and snapped to this grid.

Extracting driver data values to reef survey sites – For all raster format data sets, the ‘Extract Multi Values to Points’ tool was used to assign raster cell values based on the pixel that each reef survey fell inside. For all other data sets, each reef survey was assigned values based on the

centroid of the closest land-sea human impact or environmental factor data point using the 'Spatial Join' tool. See below for specific details on each local land-sea human impact and environmental factor dataset.

Local land-sea human impacts and environmental factors

Human Population Density – NASA Gridded Population of the World v4 (GPWv4) data is available at 30 arc-second resolution (~1 km) and represents the number of people living in each pixel. This is a global dataset and the quality of census information varies by country. For the United States, the input data to the gridding process for our region was census-block level population, which represents the finest resolution available from the US Census Bureau. The dataset is based on 2010 census population data and used county level growth rates calculated from 2000–2010 to produce gridded population data at 5-year intervals from 2000–2020.

The Island of Hawai'i was extracted from the global GPW v4 dataset and projected to UTM at 907.5 m resolution (Fig. S12). The 'Focal Statistics' tool was used to calculate the total population within 15 km of each oceanic pixel in our study region. Output rasters were clipped to cover nearshore waters from the shoreline to 5 km offshore. Linear interpolation by pixel was used to generate rasters for missing years and to produce annual time steps of human population density for years 2000–2019.

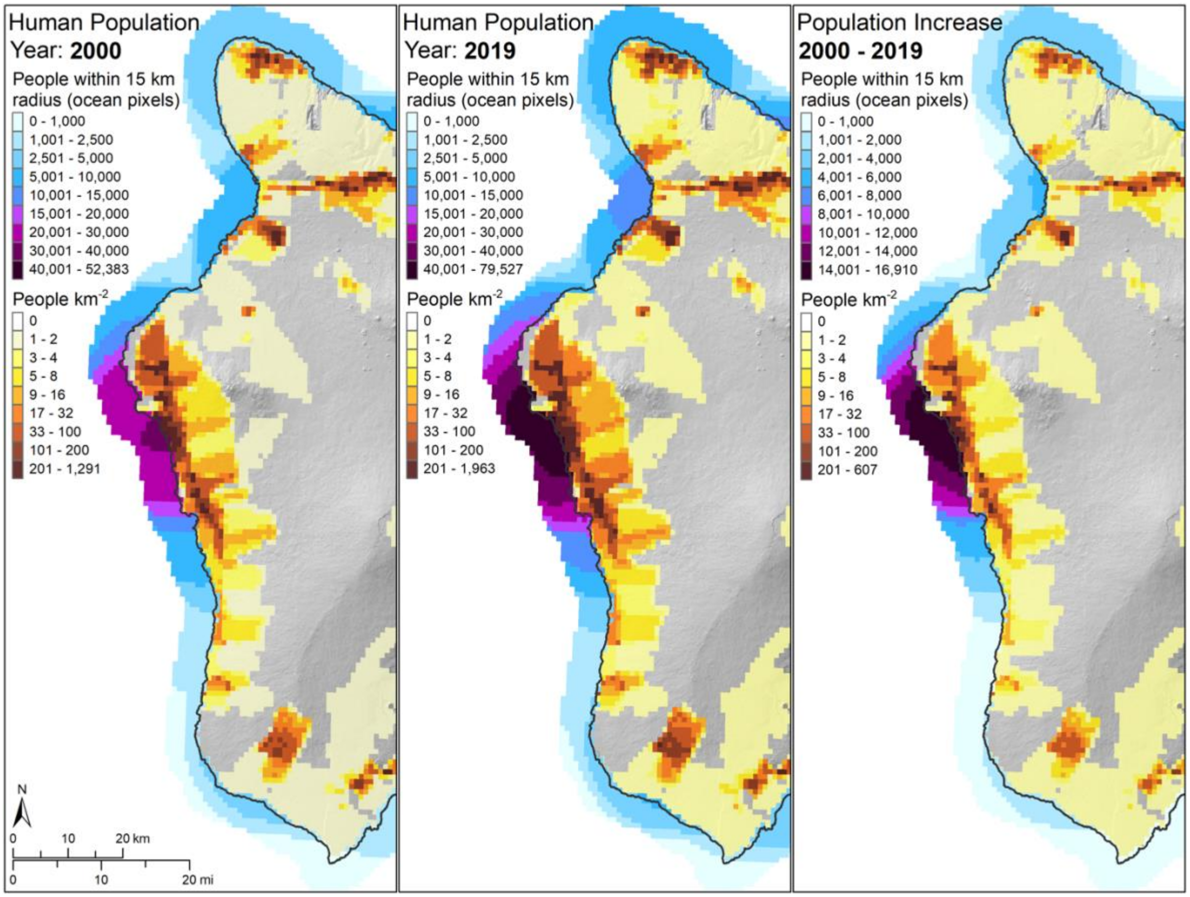

Fig. S12. Human population density on land from NASA GPW v4 and calculated data layer showing the total population within 15 km of each nearshore oceanic pixel for years 2000 (left), 2019 (middle), and increase from 2000 to 2019 (right).

Wastewater Pollution - Onsite sewage disposal systems (OSDS) (e.g., cesspools, septic tanks, aerobic treatment units) and injection wells are prevalent in Hawaiʻi, where municipal sewer infrastructure does not exist for a majority of residences. We mapped wastewater effluent and nutrient input from OSDS and injection wells into coastal waters at 100 m resolution in annual time steps from 2000–2017 across the study region (Fig. S13–S15). We produced two sets of driver layers: total effluent (L ha^{-1} yr^{-1}) and nitrogen input (kg ha^{-1} yr^{-1}). Values from 2017 were used for 2018 and 2019.

OSDS and injection well data were obtained from the Hawaiʻi Department of Health. OSDS locations are based on centroids of Hawaiʻi County Tax Map Key parcel data. OSDS and injection wells have estimated effluent and nutrient fluxes based on the number of bedrooms and bathrooms that they service and the type of system in use[23]. Injection well locations, effluent rates and nutrient concentrations were based on data extracted from the Hawaiʻi Department of Health Underground Injection Control Permit records[24]. OSDS and injection wells were assigned a date that they went into service based on available County and State records, and were updated by extracting more recent data from the Hawaiʻi Department of Health Individual Wastewater System Permit Database[25]. Nutrient loading and discharge rates were updated based on a more recent assessment for West Hawaiʻi[26]. We limited OSDS and injection wells to those within a 1-year ground water travel time of the coast, based on 3D ground water modelling with MODFLOW and MODPATH[23]. Within the 1-year travel time zone in West Hawaiʻi, there were 5,156 OSDS records (3,574 in operation in the year 2000 increasing to 4,933 in 2017). Of these, 1,049 OSDS records (20%) could not be constrained to a start date of service. Since relevant records started to be maintained in digital databases in the mid 1990's we assumed that all of these OSDS with missing dates went into service before the year 2000 (i.e., the starting time period of our study). On Hawaiʻi Island there are 32 injection wells, 5 of which fall inside the 1-year travel time zone in our study region. Wastewater effluent and nutrient loading from each OSDS and injection well were assumed to flow to the nearest point on the shoreline and then plume outward from there using a Gaussian decay function with distance from shore approaching zero at 2 km offshore (*sensu*[3]).

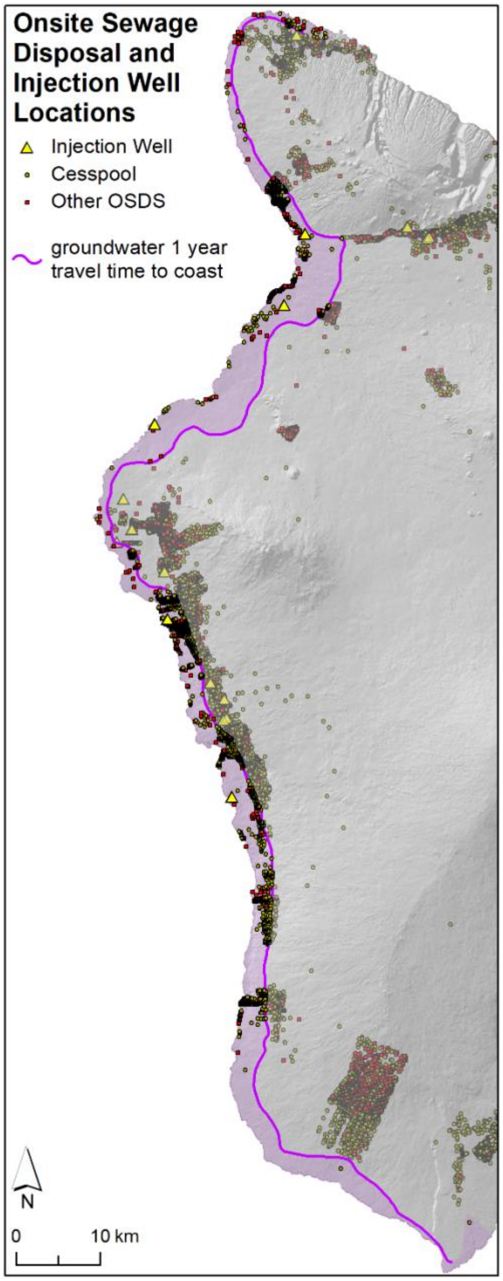

Fig. S13. Map of onsite sewage disposal systems (OSDS) and injection wells across our study region in West Hawai‘i. The purple line delineates the 1-year travel time in the groundwater to reach the coast and was used as a cut-off for inclusion of onsite sewage disposal systems (OSDS) and injection wells in this analysis.

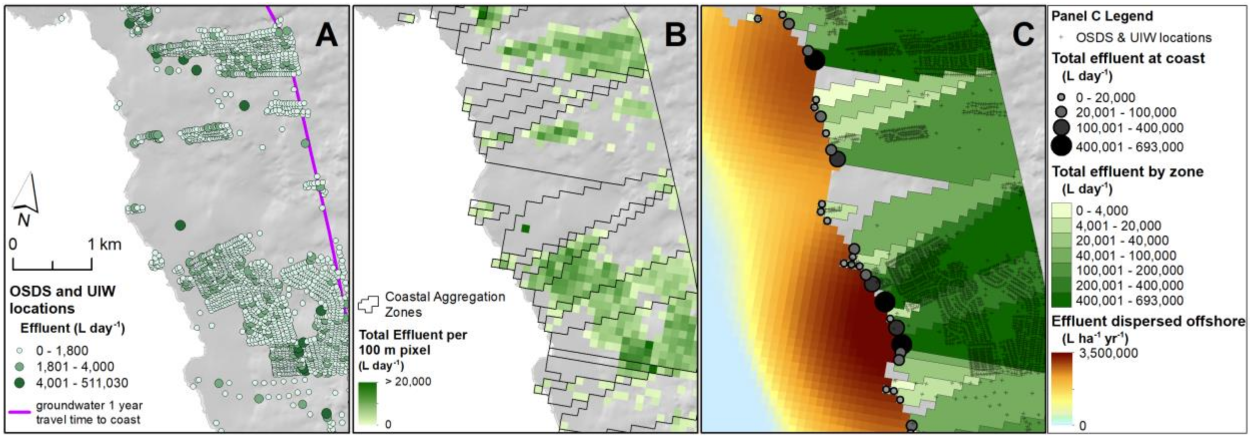

Fig. S14. These maps zoomed in to Kailua-Kona illustrate the sequential process of deriving wastewater pollution from OSDS and injection well location data. A) OSDS and injection well locations (circles) symbolised by effluent input into the environment: small, light green circles represent lower effluent and larger, dark green circles represent higher effluent. The purple line represents the 1-year travel time of groundwater to the coast. B) Total effluent from OSDS and injection wells summed within 100 m pixels, with increasing values represented by light to dark green shading. Zones connecting OSDS and injection wells to the nearest pixel on the shoreline are outlined in black. C) Zones coloured by increasing total effluent (the sum of effluent pixels shown in panel B by zone; light to dark green), overlaid with OSDS and injection well locations (small grey crosses), and the terminal point of each zone along the shoreline symbolised by increasing total effluent (light to dark grey circles of increasing size). Terminal points have identical effluent values to their corresponding zones and are used as the source features for offshore dispersal with a Gaussian decay function (light blue to dark orange shading represents low to high effluent input).

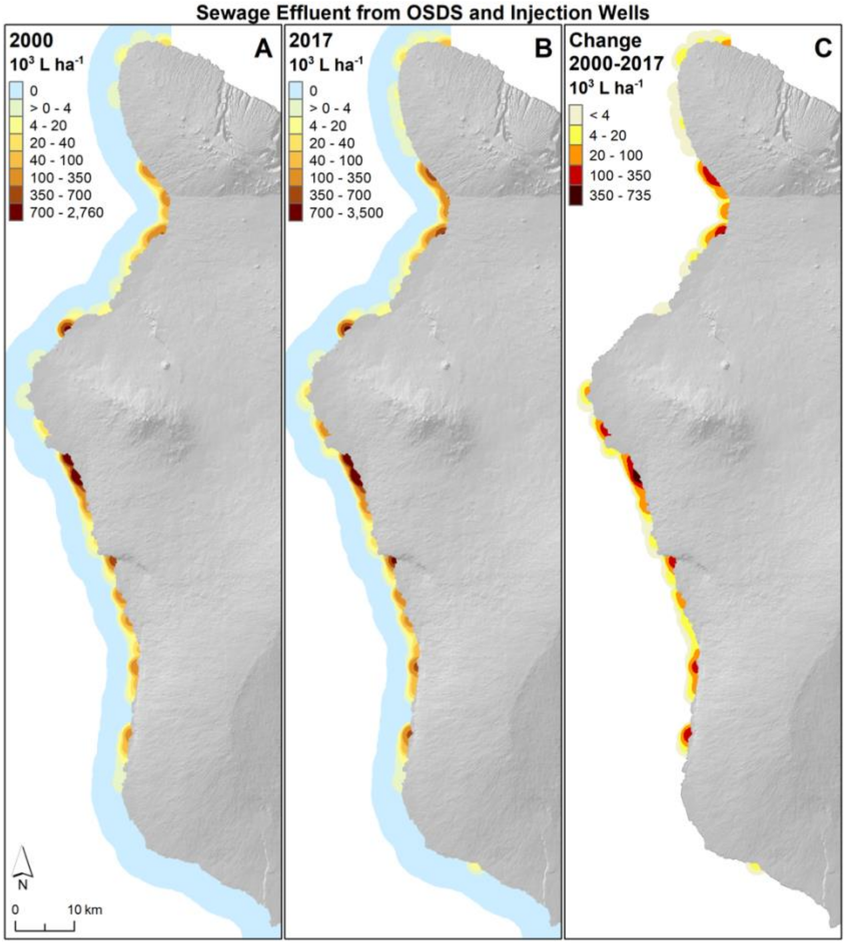

Fig. S15. Total effluent from OSDS and injection wells to coastal waters for years (A) 2000, (B) 2017, and (C) the change from 2000 – 2017. Note that the units are 1000 L ha^{-1}.

Nitrogen Input – We calculated nutrient input as the combination of total nitrogen from wastewater pollution (i.e., onsite sewage disposal systems and injection wells; Fig. S15) and golf courses (Fig. S16 – S18). See *Wastewater Pollution* section above with respect to quantifying nutrient input from wastewater.

For golf courses, we developed a series of maps representing nitrogen input from golf course runoff and leeching to groundwater at 100 m resolution for the years 2001 – 2017. The maps are based on the total area of golf course within drainage basins upslope of nearshore waters. Golf course area was derived from NOAA Coastal Change Analysis Program (CCAP) land-use/land-cover data and Landsat 7 and 8 cloud-free composite images created with Google Earth Engine. Golf course area was multiplied by a nitrogen application rate of 585 kg ha^{-1} yr^{-1} [27,28] and then by a leaching rate of 32%[29-31] to estimate flux of nitrogen that runs off or reaches the groundwater. We also imposed a decrease in the amount of nutrients reaching the coast with increasing distance inland according to the following logic: golf courses < 5 km from the coastline, we assume 100% of leached nutrients reach the ocean; from 5–10 km the proportion of nutrients reaching the coast decreases linearly from 100–0%; no golf courses are located beyond 10 km from the coast. We used sub-watershed catchment polygon data from USGS Stream Stats[32] to estimate nutrient transport from golf courses to the coastline. We then evenly distributed nutrients along the coastal line segments of drainage polygons and used those as the source features to plume nutrients offshore using a Gaussian decay function with distance from shore (Kernel Density tool), approaching zero at 2 km offshore (*sensu*[3]).

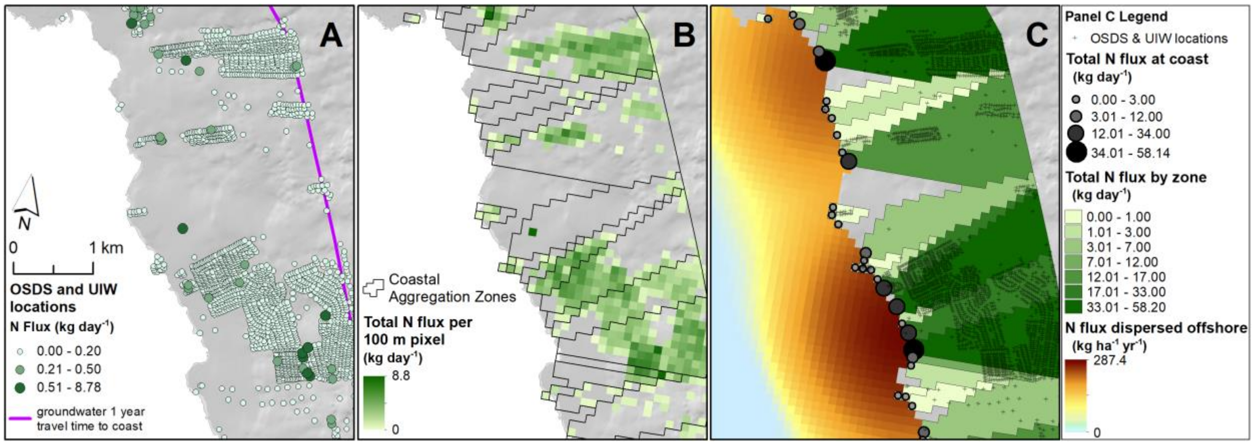

Fig. S16. Maps zoomed in to Kailua-Kona illustrate the sequential process of deriving the wastewater nitrogen input from point source OSDS and injection well location data. A) OSDS and injection well locations (circles) symbolised by nitrogen input into the environment: small, light green circles represent lower nitrogen input and larger, dark green circles represent higher nitrogen input. The purple line represents the 1-year travel time of groundwater to the coast. B) Total nitrogen input from OSDS and injection wells summed within 100 m pixels, with increasing values represented by light to dark green shading. Zones connecting OSDS and injection wells to the nearest pixel on the shoreline are outlined in black. C) Zones coloured by increasing total nitrogen input (the sum of N flux pixels shown in panel B by zone; light to dark green), overlaid with OSDS and injection well locations (small grey crosses), and the terminal point of each zone along the shoreline symbolised by increasing total nitrogen input (light to dark grey circles of increasing size). Terminal points have identical nitrogen flux values to their corresponding zones and are used as the source features for offshore dispersal with a Gaussian decay function (light blue to dark orange shading represents low to high nitrogen input).

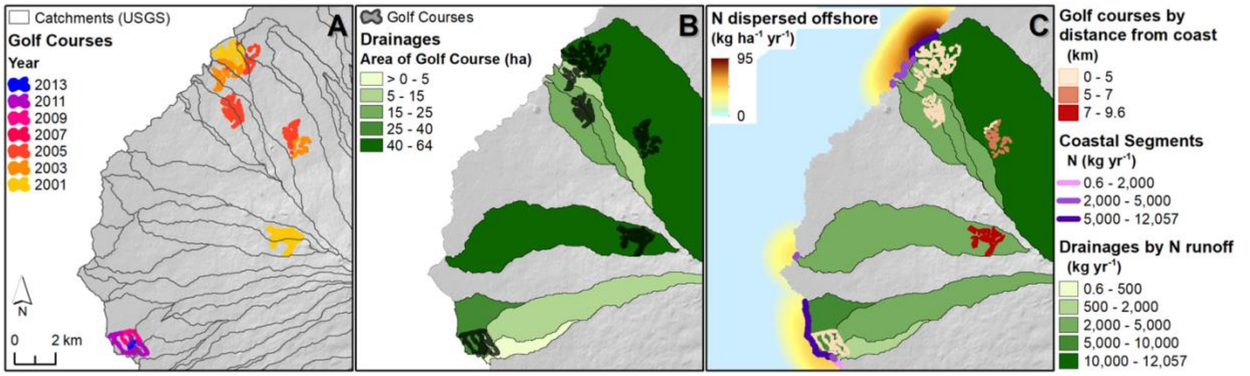

Fig. S17. Maps zoomed in to Keāhole Point vicinity illustrate the sequential process of deriving golf course nitrogen runoff from golf courses. A) Golf course footprints extracted from satellite imagery, coloured by the first year in which they appear in our time series of Landsat cloud free composite images (yellow to red to blue colouring), overlaid with USGS sub-watershed catchments (grey lines). B) Catchment polygons aggregated into larger drainage polygons that reach the coastline, coloured by the area of golf course contained in each (increasing from light to dark green). Golf course footprints are shown in dark grey. C) Drainages coloured by estimated nitrogen from golf courses reaching the coast (light to dark green) based on nitrogen application rate, leaching rate, and distance inland from coast. Coastline segments of each drainage are symbolised by the nitrogen load of the corresponding drainage (increasing from light to dark purple) and used as the input source features for offshore dispersal by a Gaussian decay function (light blue to dark orange shading represents low to high nitrogen input). Golf course footprints are coloured by distance inland from coast (increasing from light beige to red).

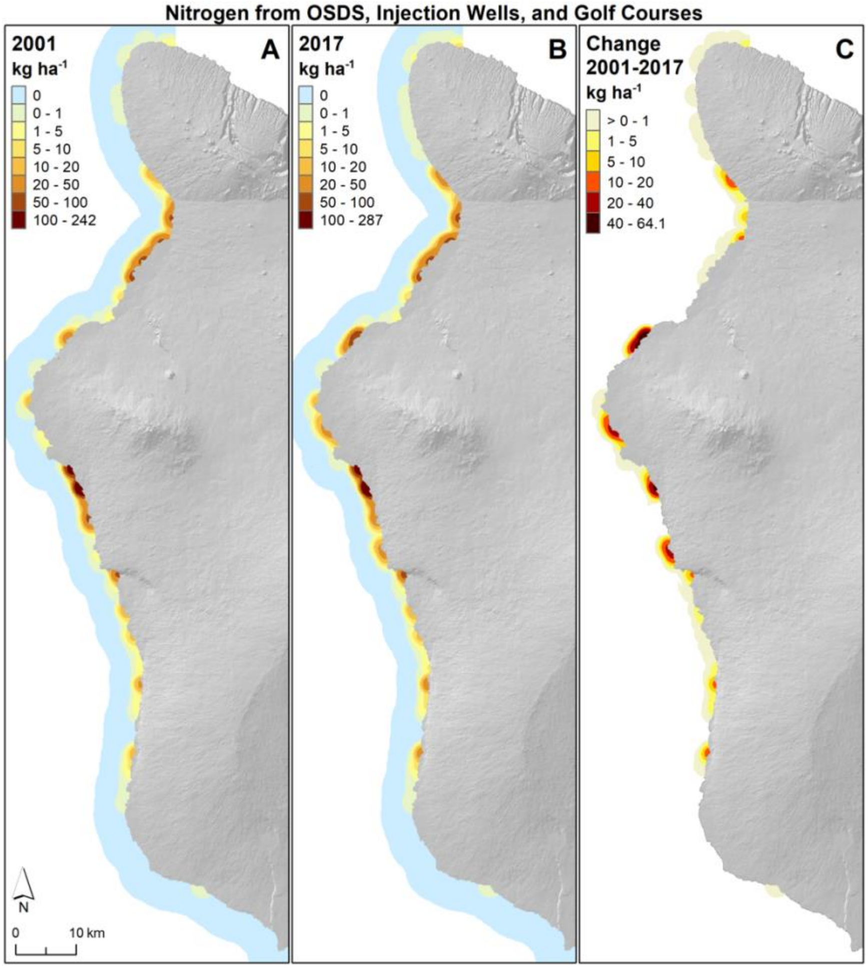

Fig. S18. Combined nitrogen input from OSDS, injection wells, and golf courses into coastal waters for years (A) 2001, (B) 2017, and (C) the increase in nitrogen input from 2001 – 2017.

Urban Runoff – We used the total area of impervious surfaces (e.g., paved roads, car parks, pavements, and roofs) as a proxy for urban runoff into the ocean that includes household chemicals, oils, trash, sediments, and other pollutants. We derived proxy layers for the impact on nearshore reefs based on the total area of developed impervious surface within 10 km of the coastline by watershed at 100 m resolution (Fig. S19–S20). We extracted impervious surface cover from NOAA CCAP land use land cover data from 1992, 2001, 2005, and 2010. We also digitised 2017 impervious surface cover from a single cloud-free Landsat 8 image (30 m resolution, 15 m pan-sharpened). We used sub-watershed catchment polygon data from USGS Stream Stats[32] to aggregate the impervious surface area by drainage to coastal source pixels and plumed the values offshore from there using a Gaussian decay function with distance from shore (Kernel Density tool), approaching zero at 2 km offshore (*sensu*[3]). CCAP data is available as follows: CCAP 30 m resolution is available from 1992–2005 and CCAP 2.4 m resolution from 2005 and 2010 We aggregated 2005 and 2010 high resolution impervious surface to 28.8 m resolution, the closest approximation to 30 m without resampling (2.4 x 12 = 28.8), before processing. Aggregated 2010 impervious surface was used as the starting reference point for digitizing 2017 land cover, which was subsequently converted to raster at 28.8 m resolution. For 2005, there are both high resolution (2.4 m) and regional (30 m) CCAP products available. We took advantage of this by running the offshore dispersal of impervious surface area from both 2005 products and created a spatial calibration factor raster based on the ratio of the two layers. We multiplied the 2010 and 2017 data by this calibration factor so that impervious surface change is consistent and comparable across time steps. We filled missing years by linear interpolation across years to produce annual maps. Values from 2017 were used for years 2018 and 2019.

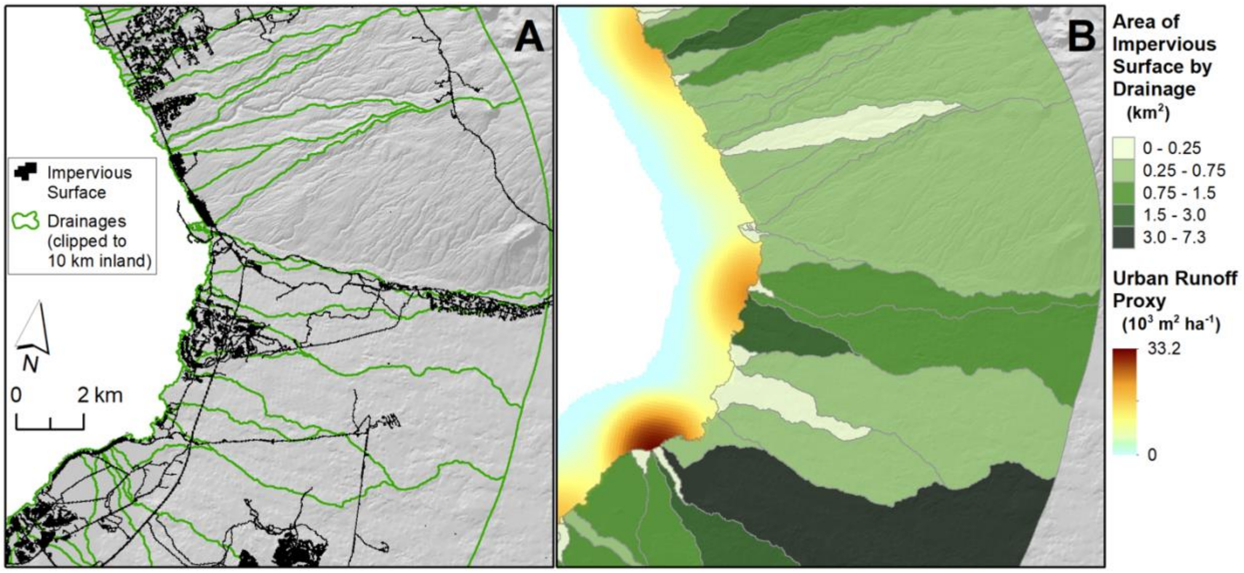

Fig. S19. These maps zoomed in to Kawaihae Bay area illustrate the process of deriving our proxy layer for urban runoff from impervious surface land cover data. A) Impervious surface land cover footprint (black) for an example year overlaid with sub-watershed drainage polygons clipped to 10 km inland. B) Drainage polygons coloured by area of impervious surface (light to dark green), and dispersal offshore by Gaussian kernel decay function (light blue to dark orange shading represents low to high urban runoff).

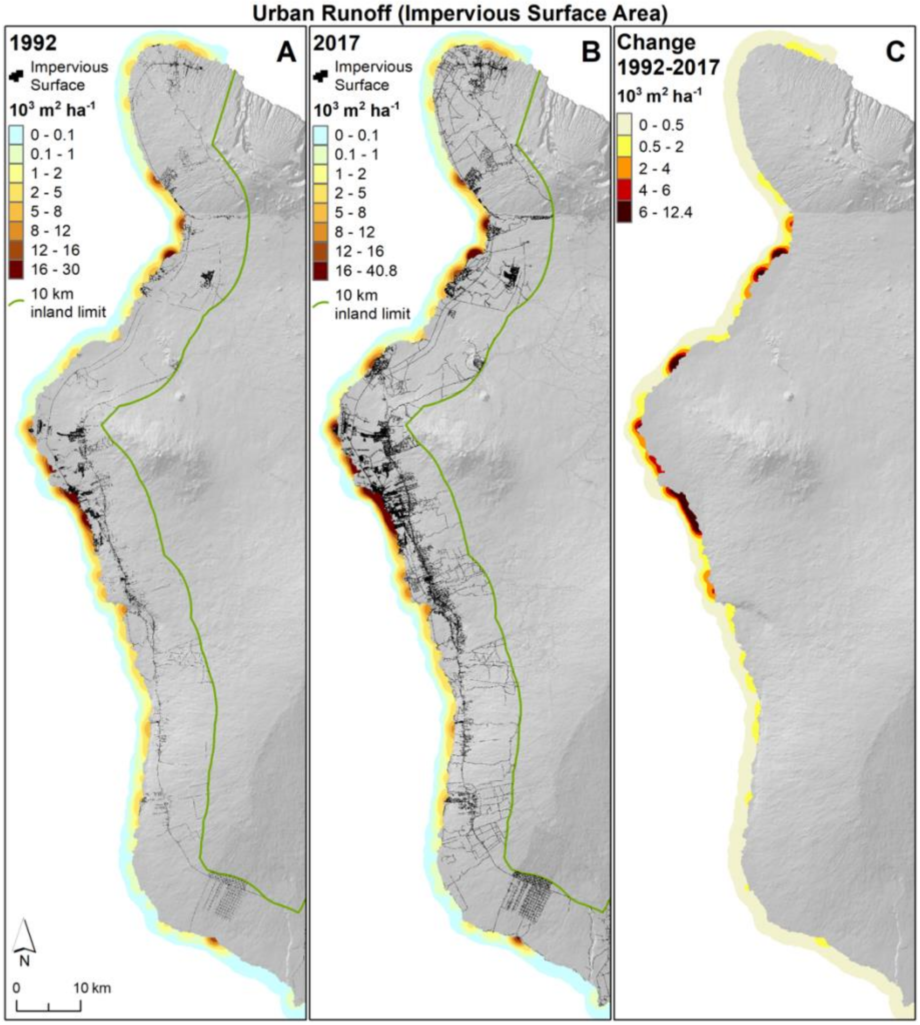

Fig. S20. Maps showing the impervious surface land use footprint on land (black) within 10 km inland of the shoreline (green line), and derived proxy for urban runoff into coastal waters for years (A) 1992, (B) 2017, and (C) the increase from 1992–2017.

<u>*Rainfall*</u> – We quantified annual time steps for two metrics of rainfall – annual rainfall and peak rainfall – from 1990–2019. Data for daily rainfall in mm by rain station was compiled, gap-filled, and updated with support of Ryan Longman (University of Hawai‘i)[33,34]. We filtered rain stations and only included those that had at least 80% of daily observations for each year (Fig. S21). The annual rainfall and peak rainfall (i.e., maximum 3-day rainfall total) at each station was calculated and used to derive interpolated surfaces of each metric by year with Empirical Bayesian Kriging in ArcGIS (See Fig. S24A for example). USGS ArcHydro-derived subwatersheds[32] clipped to 0–10 km from the coast were used to calculate total annual and peak rainfall per drainage area. The resulting values were dispersed offshore from coastal line segments of watersheds using a Gaussian decay function with distance from shore, approaching zero at 2 km offshore (Fig. S22). The final rainfall driver layers are 100 m resolution.

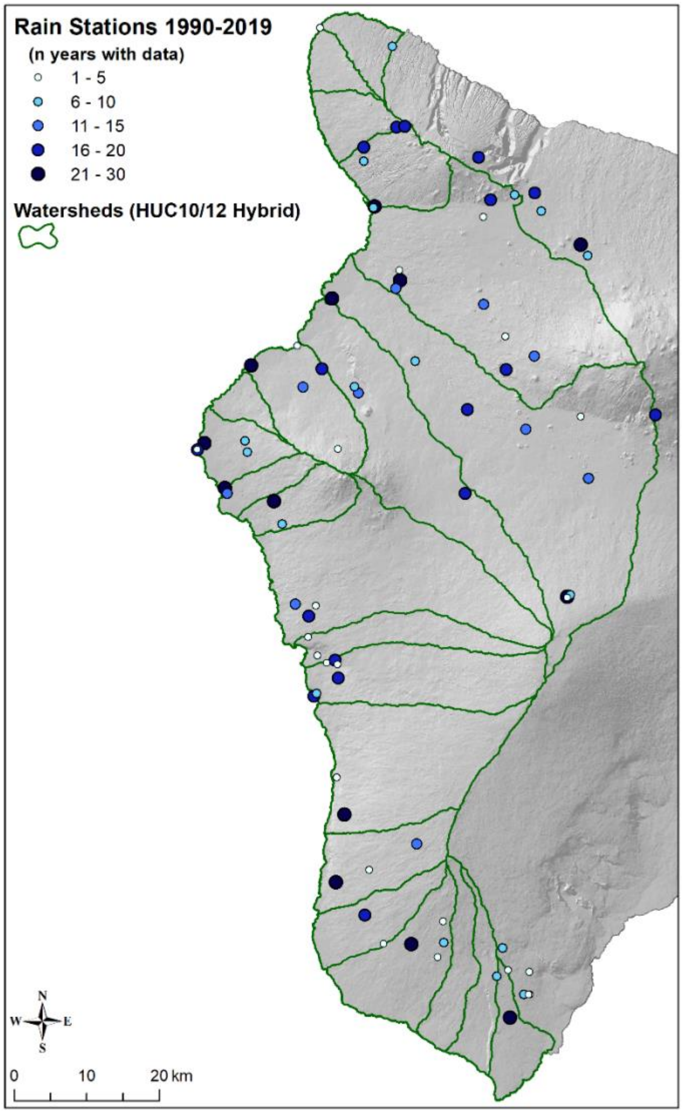

Fig. S21. Locations of rain stations used in this project symbolised by number of years with sufficient data to be included, and watershed boundaries (a hybrid of USGS WBD HU10 and HU12 data).

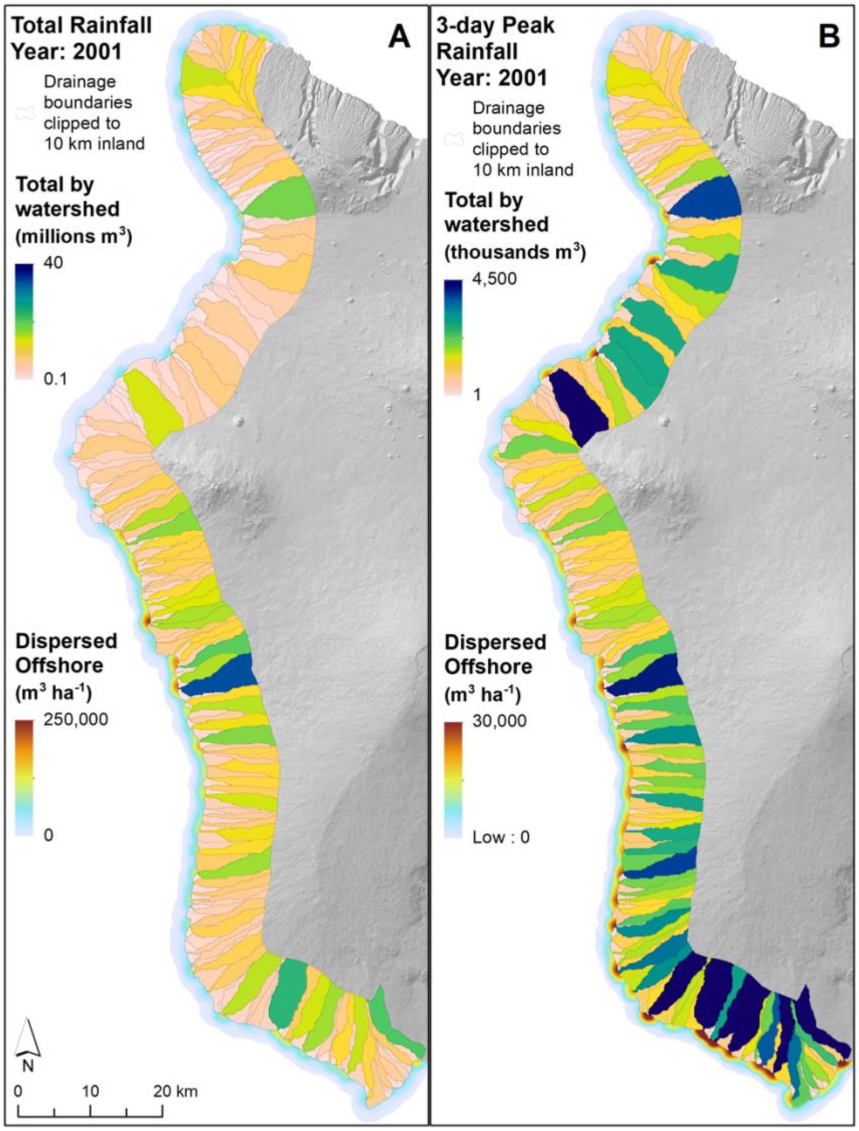

Fig. S22. Map showing the annual total (A) and peak 3-day total (B) rainfall for example year 2001. Totals from interpolated rainfall by watersheds clipped to 10 km inland are shown on land, and dispersed offshore. Layers are symbolised relative to the range in values over the full 30-year time series (1990–2019). In 2001, the highest peak rainfall occurred in Kaʻū (far southern coastline) as well as south Kohala, while total rainfall was moderate to low for most of the study area. In many years there are more similar spatial patterns between the two metrics with the same areas experiencing both high total and peak rainfall.

Sediment Input – Here we quantified sediment input to nearshore waters at annual time steps from 1990 – 2019 at 100 m resolution (Fig. S23 – S24). We used the Integrated Valuation of Ecosystem Services and Tradeoffs (InVEST) sediment delivery model to derive long-term annual average sediment input reaching the coast[35-37]. We then dispersed coastal sediment values offshore (sensu[3]). This long term annual average sediment input dataset was modulated over time by watershed based on storm discharge calculated from daily rainfall data updated through 2019 and fully gap-filled[33,34] (see *Rainfall* above). We calculated the annual maximum 3-day rainfall total for each rain station location (‘peak rainfall’), then produced interpolated surfaces for each year and summarized them by a hybrid of HUC10/HUC12 scale watersheds from the USGS National Hydrography Dataset. The USDA Natural Resources Conservation Service (NRCS) methodology for calculating direct runoff of rainfall based on storm rainfall[38] was used to calculate discharge from the peak rainfall event for each watershed in each year. The curve number (CN) raster used to calculate discharge was derived from 2010 CCAP data, gSSURGO soil hydro group data, and the NRCS handbook[39]. Sediment load was assumed to scale with

discharge according to approximate ratings curve as follows[40]: sediment = a x Q^2. Where Q is storm water discharge volume, and a is a calibration factor. A weighting factor was calculated for each watershed-year based on squared discharge and then multiplied by the annual average sediment layer. This works out such that taking the average of all annual sediment time steps will reproduce the original InVEST-based average annual sediment layer, and the sum of all annual sediment time steps will produce the average annual sediment layer multiplied by n years.

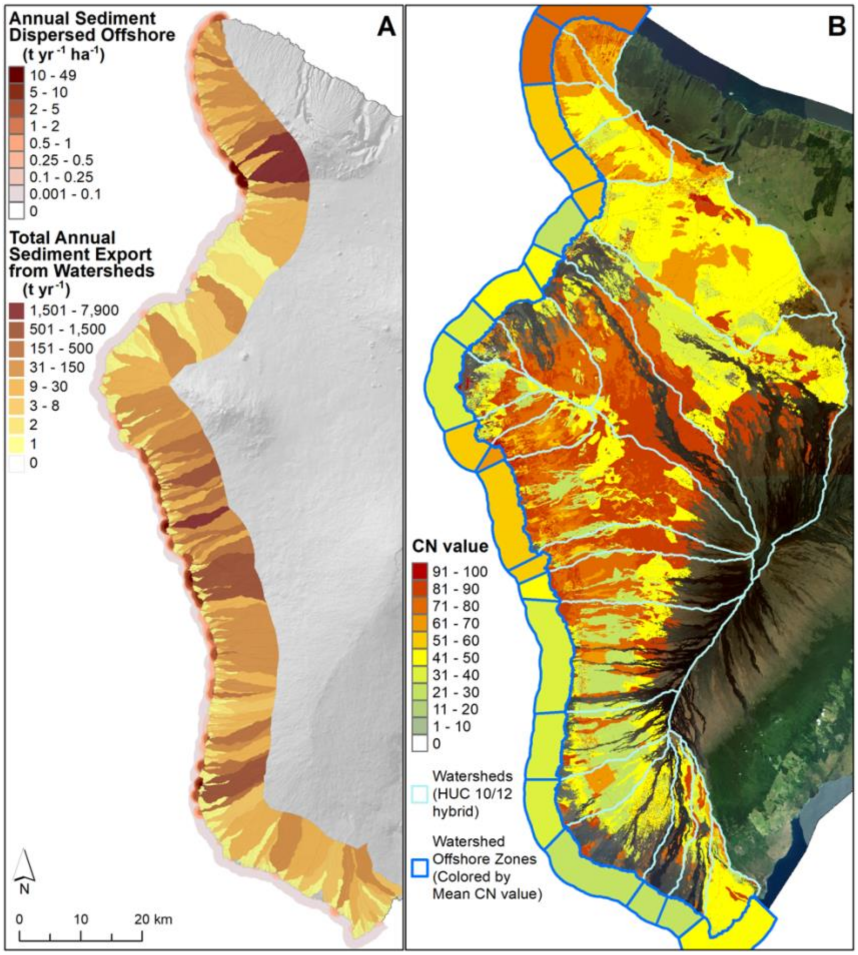

Fig. S23. Maps showing the static data input components used to produce the annual time series of sediment input. A) Average annual sediment load in tons by subwatershed (light yellow to brown; from 0-10 km inland), and average annual sediment load dispersed offshore with Gaussian kernel decay function (light pink to dark red). All values were derived from InVEST sediment delivery modelling. B) Curve number (CN) raster used in calculation of surface runoff (discharge) from rainfall data. CN values represent the runoff potential of hydrologic soil-cover complexes. Low values of CN represent low runoff potential and occur in highly permeable areas where the ground can absorb large amounts of rainfall before surface runoff occurs. High values of CN reflect areas where very little rainfall is required before surface runoff occurs (i.e., high runoff potential). The CN raster was derived by combining high resolution NOAA CCAP data (2010) and USGS soil hydro-group data (gSSURGO). CN values were assigned following the NRCS hydrology handbook[26]. Note that CN values of zero are displayed as no-colour and therefore the satellite image background shows through in these areas.

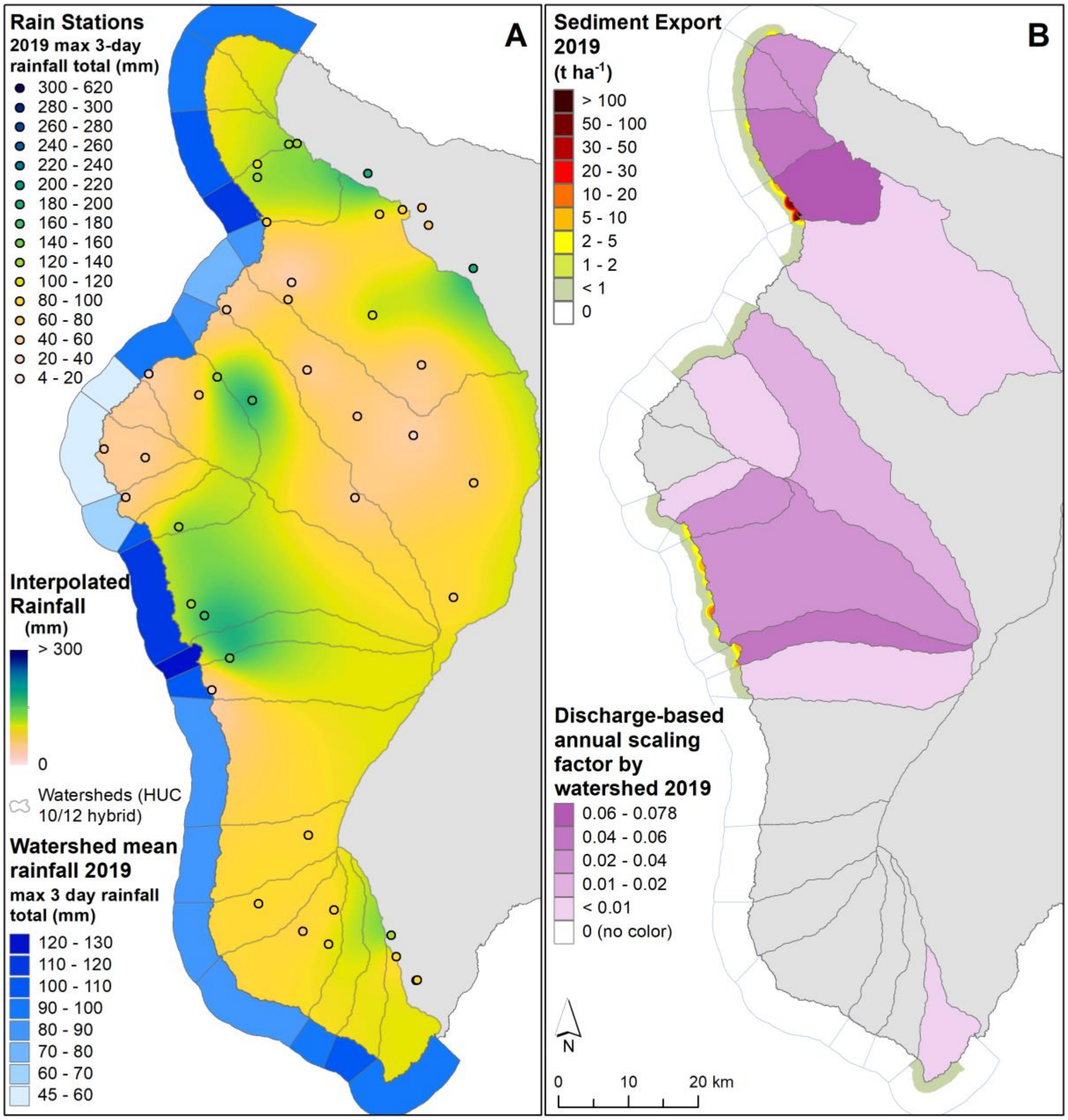

Fig. S24. Maps showing intermediate steps and final output of sediment pollution for example year 2019. A) Rain station locations (circles) coloured by 2019 observed peak rainfall and interpolated 2019 peak rainfall surface with comparable symbology to rain station points (light beige to yellow to green), watershed boundaries (grey lines), and 2019 means of peak rainfall by watershed shown by offshore watershed zones (light to dark blue shading). Watershed-scale peak rainfall means are then combined with watershed-scale average CN values to calculate storm water runoff by watershed. B) Annual scaling factor by watershed (light to dark pink shading; the 2019 proportion of the 30-year total peak 3-day storm discharge squared, for each watershed), and the final sediment export driver layer for year 2019 (light green to dark red shading nearshore along the coast). Watersheds with a value of zero (no colour) did not receive a large enough peak rainfall event to generate sediment runoff in the given year.

Fishing Gear Restrictions – Fishing regulation information and marine managed area (MMA) boundary designations were obtained from[3]. All MMA regulations were evaluated for whether the following gear categories or types of fishing were allowed that target reef fin fish: line fishing, lay nets, spear fishing, and aquarium collection. Areas that are explicitly full no-take with regard to reef fin fish were also recorded as such. We summarised the existing regulations into six categories for our study region (Table S1).

Table S1. Categories of fishing gear restrictions based on whether different gears or types of fishing that target reef fin fish only are allowed in each area across our study region.

**Gear Category**	**Rank**
Full No-Take	1
No Lay Net, No Spear, No Aquarium	2
No Lay Net, No Aquarium	3
No Lay Net	4
No Aquarium Collection	5
Open to all gears	6

We tracked changes to fishing gear regulations over time from 2000–2019. We consulted Hawaiʻi Division of Aquatic Resources (DAR) staff, news articles about regulation changes and MMA establishment, and historical versions of relevant Hawaiʻi Administrative Rules accessed via the Internet Archive Wayback Machine.

Below is a brief summary timeline of changes through time in fishery gear regulations across West Hawaiʻi:

1. December 31, 1999: West Hawaiʻi Regional Fisheries Management Area established consisting of 9 Fish Replenishment Areas (FRAs), that ban aquarium collection.
2. August 1, 2005: Four new Netting Restricted Areas (NRA; no lay nets) established plus lay net ban rules extended to 2 of the FRAs. In addition, lay netting was restricted in waters deeper than 24.4 m (80 ft) for all of West Hawaiʻi.
3. December 26, 2013: A new FRA was established at Kaʻohe (‘Pebbles Beach’) and aquarium ban rules were officially extended to most existing MMAs and FMAs. There were also several new region-wide rules that went into effect including: No SCUBA Spear; No take of sharks and rays; No aquarium take except for an established list of species by DAR (i.e., ‘White List’ species).
4. July 29, 2016: Kaʻūpūlehu Marine Reserve established a no-take MMA within the existing boundaries for areas that are shallower than 36.5 m depth (~120 ft).

We derived multiple GIS layers of fishing gear regulations corresponding to dates of rule changes by location. To assign fishing gear rank to each reef survey site, we subset the survey data by date and used the Spatial Join tool with the fishing gear layer corresponding to the dates in the subset. We used the exact position coordinates of each reef survey site to determine whether a site was inside of an MMA. We then used a 25 m search distance to assign values for the most restrictive fishery regulations for each site, as in some cases reef surveys fell within overlapping MMAs. The search radius was included following the logic that reef surveys represent 25 m transects with positional accuracy of +/-10 m. Therefore, a survey point that fell

just outside a given MMA boundary may represent a survey that was actually inside or at least partially inside the MMA boundaries. See Fig. S25 for the spatial distribution in fishing restrictions along West Hawai‘i.

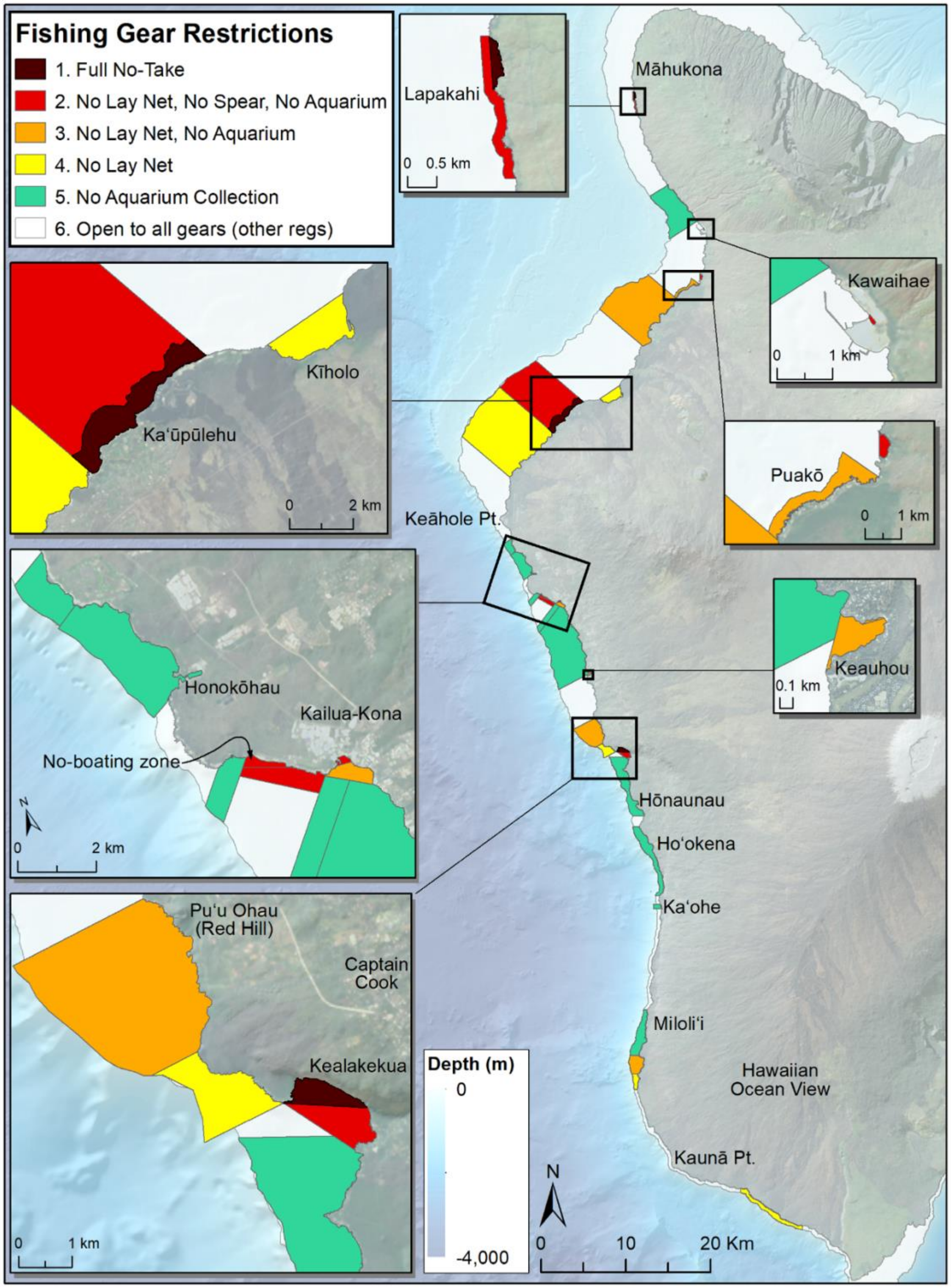

Fig. S25. Map showing fishing gear restrictions and corresponding rank from highly restrictive to open to all gear types (1 to 6, respectively), for each gear restriction category in West Hawai‘i.

Sea Surface Temperature – We used daily 5-km resolution sea surface temperature data obtained from NOAA's Coral Reef Watch from 2000–2019. The summertime mean (SST mean) and summertime variability (SST variability) SST metrics were obtained by calculating the mean and standard deviation over a 90-day window centred on the maximum value of the 7-day moving window average for each SST pixel. The regional temperature data (Fig. 3a) was calculated by taking the mean of all coastal pixels across our study region for each time step. All pixels that directly overlapped with land were removed prior to assigning values to reef survey sites. Note that 2019 was excluded in the OLR analysis owing to the marine heatwave that impacted Hawai'i[17], but occurred after our 2019 fish and benthic surveys. All data were NOAA's Coral Reef Watch 5 km Coral Temp v3.1 data set (https://coralreefwatch.noaa.gov/product/5km)[41]. See Fig. S26 for the long-term mean in SST metrics across the study region.

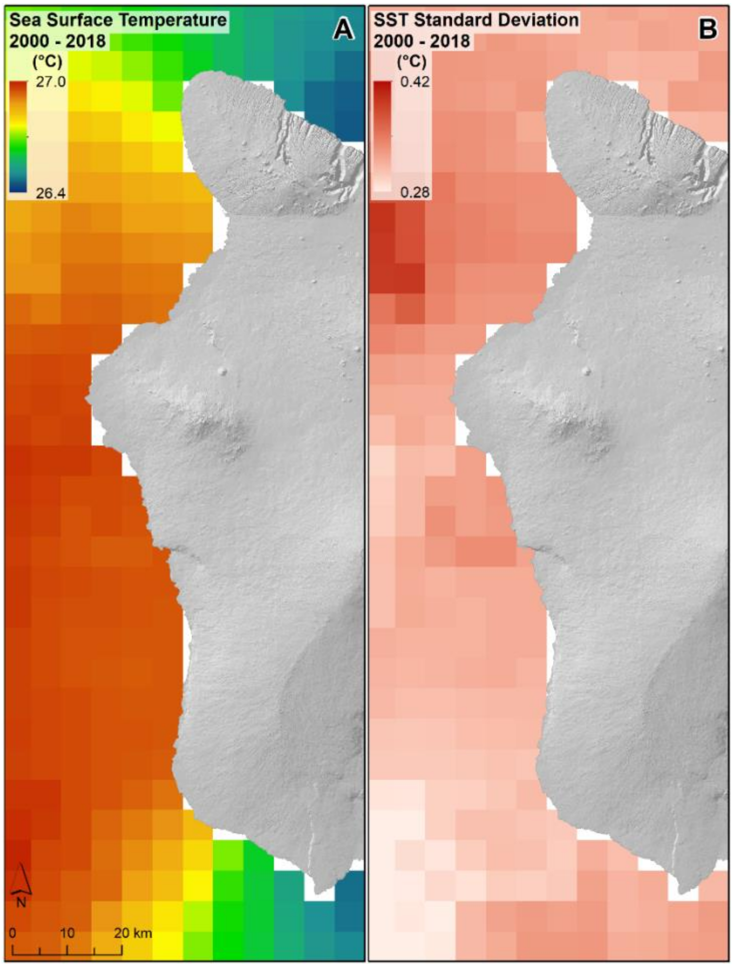

Fig. S26. Map showing the mean (A) and standard deviation (B) in summertime SST across the study region. Maps show the long-term mean from 2000–2018.

Heat Stress – We explored numerous metrics to quantify the heat stress endured by reefs across our study region during the 2015 marine heatwave. All values were calculated over the July – November 2015 period. All data were NOAA's Coral Reef Watch 5 km Coral Temp version 3.1 data set[41]. Note that the bleaching threshold is a pixel specific value calculated as 1°C above the climatological maximum monthly mean.

- Degree Heating Week (DHW): represents the accumulation of heat stress above the bleaching threshold over a 12-week period. DHW is the dominant metric in coral reef research to quantify heat stress on corals.
- Maximum HotSpot: represents the maximum daily temperature above the bleaching threshold during the marine heatwave.
- Maximum SST Anomaly: represents the maximum daily temperature above the climatological maximum during the marine heatwave.
- Maximum SST: represents the maximum daily temperature during the marine heatwave.

Ultimately, we found that all of the above heat stress metrics were highly correlated ($r > 0.8$). We therefore used DHW as the preferred heat stress metric given that it is widely understood and used by coral reef scientists across the world.

Ocean Colour – Satellite derived chlorophyll-a (mg m^{-3}) – a proxy for phytoplankton biomass – was obtained from two sources (Fig. S27). We used the maximum climatological mean from 2002–2013 of 8-day, 4 km data obtained from[3]. These data were used to quantify differences in phytoplankton biomass between reefs with positive versus negative trajectories pre-disturbance (Fig. 2d, Extended Data Fig. 3).

For all subsequent figures and analyses presented in the paper, we used VIIRS (Visible Infrared Imaging Radiometer Suite) provided by NOAA's Coral Reef Watch (https://coralreefwatch.noaa.gov/product/oc/index.php). This satellite, which provided ocean colour data starting in 2014, offered much higher spatial (750 m) and temporal (daily) resolution ocean colour information for assessing values of chlorophyll-*a* and irradiance for coral reefs (Fig. S27b). As part of the GAMM analysis in which we assessed the factors that modified coral response to the marine heatwave, we took the mean for each pixel for all of 2015 and for only the months spanning the marine heatwave (July–November 2015). For the ordinal logistic regression analysis in which we assessed the factors that best explained variation in reef-builder cover 4 years following the marine heatwave, we took the mean of each pixel from 2016–2019. However, owing to the high cloud cover, numerous days along various areas across our study region contained no data during these time periods.

To account for the unequal data availability owing to clouds, poor data quality, or other factors, we first set a threshold of data exclusion: all pixels that contained less than 1 retrieval per week on average were removed. We then calculated bootstrapped means (resampled with replacement 10,000 times) of chlorophyll-*a*/irradiance for each pixel for each time period. The maximum sample number used as input for each of the 10,000 bootstrapped means represented 90% of the threshold of 1 retrieval $week^{-1}$, or ~12% of data available for each pixel. Limiting data inclusion for each bootstrapped mean ensured that all means for each pixel were based on the same sample number. All ocean colour data were masked to account for optically shallow waters (sensu[42]).

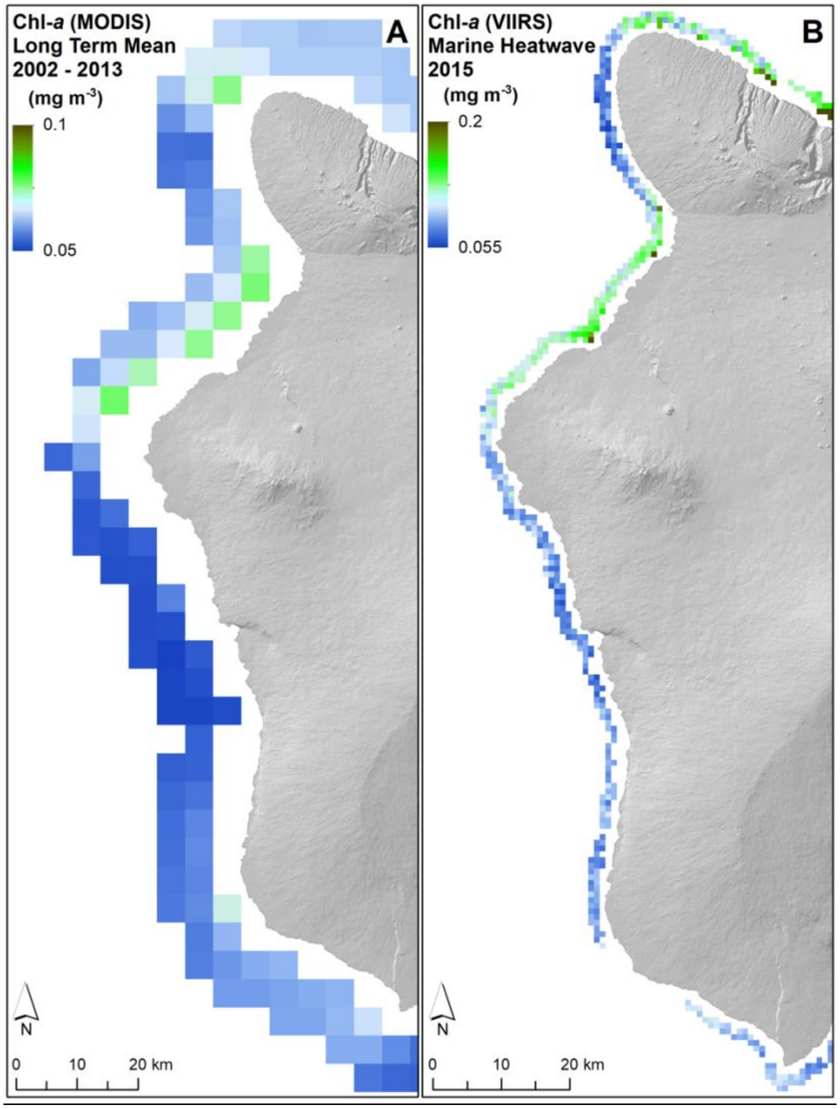

Fig. S27. Map showing the phytoplankton biomass (i.e., chlorophyll-*a*) from A)[3]. (B) VIIRS. The VIIRS data provide much higher spatial resolution (750 m *versus* 4 km) but are only available from 2014 onwards. Example year of 2015 shown.

Wave Exposure – The main Hawaiian Islands have a complex wave climate including persistent trade wind seas, year around south swells, and seasonal north swells as well as waves from tropical and subtropical cyclones passing nearby[43]. Due to its southeastern location within the Hawaiian archipelago, West Hawaiʻi is generally protected from northerly swells and therefore does not experience the annual peak in wave forcing similar to that of Kauaʻi, Oʻahu and Maui[43]. Nevertheless, we include wave exposure as a predictor in our analyses given that spatial and temporal gradients in wave forcing were present within the study period (Fig 1c, Fig. S28).

The multi-modal sea states varying with time and space are characterised by wave height, period, and spectrum. Wave power determined by these three factors provides a good representation of the wave exposure at reef sites. Due to the lack of direct buoy measurements, we obtain wave power for the west of Hawaiʻi Island from a hindcast dataset generated by the numerical wave model system assembled by[44]. The system comprises the third generational spectral wave models WAVEWATCH III[45] and SWAN (Simulating Waves Nearshore)[46] on a suite of nested grids from globe to nearshore as shown in Fig. S28. The regional WAVEWATCH III model covers

the major Hawaiian Islands with 3 arcminute resolution (~5.5 km). It is nested within the global WAVEWATCH III model from 77.5° S to 77.5° N with 0.5 arc-degree resolution and in turn provides the directional wave spectra as boundary conditions for the SWAN grids for Oʻahu, Kauaʻi, Maui, and Hawaiʻi Island with 18-arcsecond resolution (~550 m). The island-scale SWAN model can resolve the wave transformation over the insular shelf and further defines the boundary conditions for the nested SWAN model covering west of Hawaiʻi Island at 9-arcsecond resolution (~250 m). This provides a transition for the nearshore SWAN grids and allows for a detailed description of the nearshore waves with 1.8-arcsecond resolution (~50 m).

Accurate wave modelling around the Hawaiian Islands requires high-quality global and regional wind datasets. The NOAA NCEP Climate Forecast System Reanalysis (CFSR) produces assimilated surface winds at 0.5° resolution around the globe from 1979–2011[47] and 0.205° afterward[48] that provides the boundary conditions for downscaling of Hawaiʻi regional winds by the Weather Research and Forecasting (WRF) model[49,50] CFSR and its regional WRF downscaling provide good descriptions of synoptic and mesoscale weather patterns for modelling of waves from local and distant sources[44]. The surface winds from both datasets are concatenated to provide input for the numerical wave model system. The model output includes significant wave height, peak period, and peak direction as well as wave power at each grid point in hourly intervals for 1979–2019. The hindcast dataset was thoroughly validated with available measurements from offshore and nearshore buoys and satellite altimeters. The validated hindcast allows characterization of interannual and long-term wave climate as well as spatial variation of the wave conditions across the Hawaiian Islands.

We computed wave power at annual time steps from 2000–2019 at 50 m resolution (Fig. S28). Wave power (kW m^{-1}) combines wave height and period and provides a more representative metric of wave exposure than wave height alone[51]. Annual data were generated for each 50 m grid cell by taking the mean of the top 97.5% in daily maximum wave power data obtained from[44] and updated for this study.

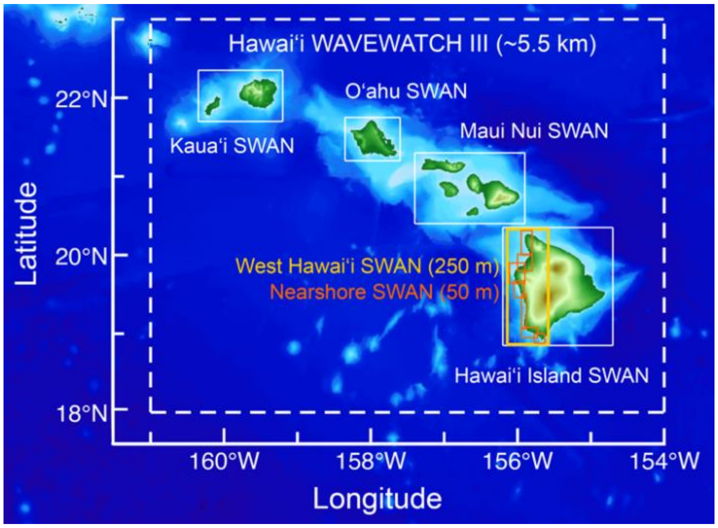

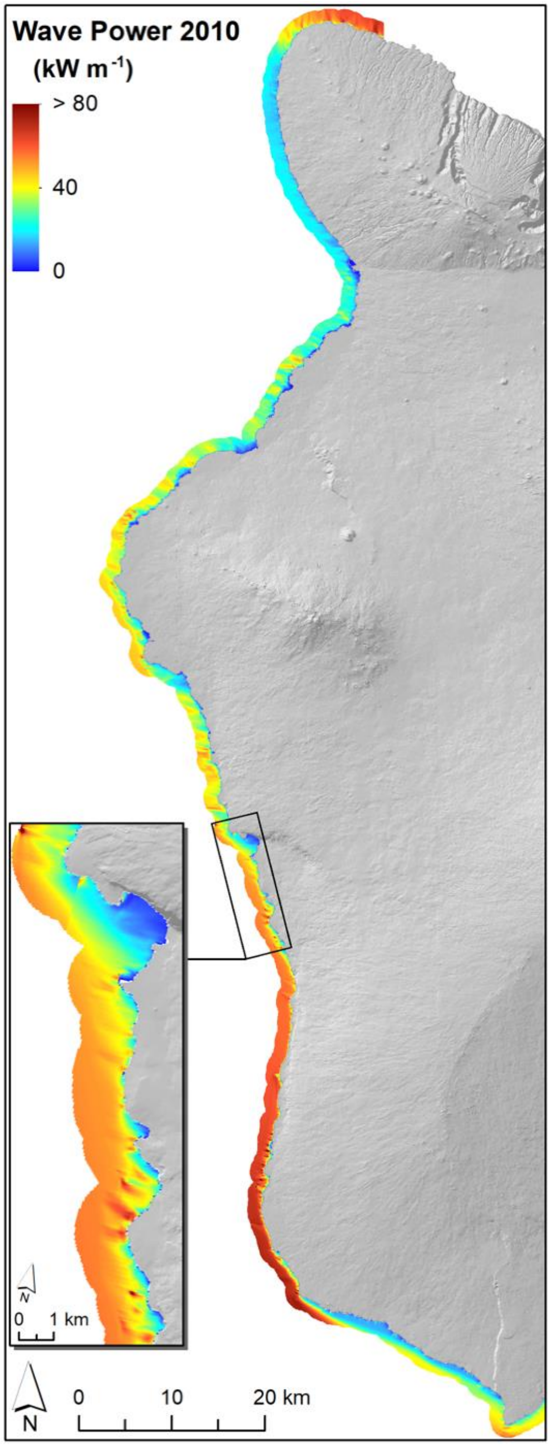

Fig. S28. Map of West Hawaiʻi showing the high resolution (50 m) wave model used in our data analysis (right). Wave power (kW m^{-1}) data are available annually from 2000–2019; 2010 is shown as an example. Computational grids of WAVEWATCH III and SWAN models in Hawaiʻi region (above). The approximate grid resolution is listed in parenthesis for each model.

Coastal Sectors – Approximately 100 m width sector polygons were derived along the entire coast of West Hawai‘i that span from the shoreline to encompass all nearshore reef area (Fig. S29). These sectors were used to extract all local land-sea human impact and environmental factor data from the 10 m depth contour (Fig. 1c). Sector Polygons were created using ArcGIS ArcPy adapted from a script by Andrew Davies, University of Rhode Island (https://github.com/marecotec/dsmtools).

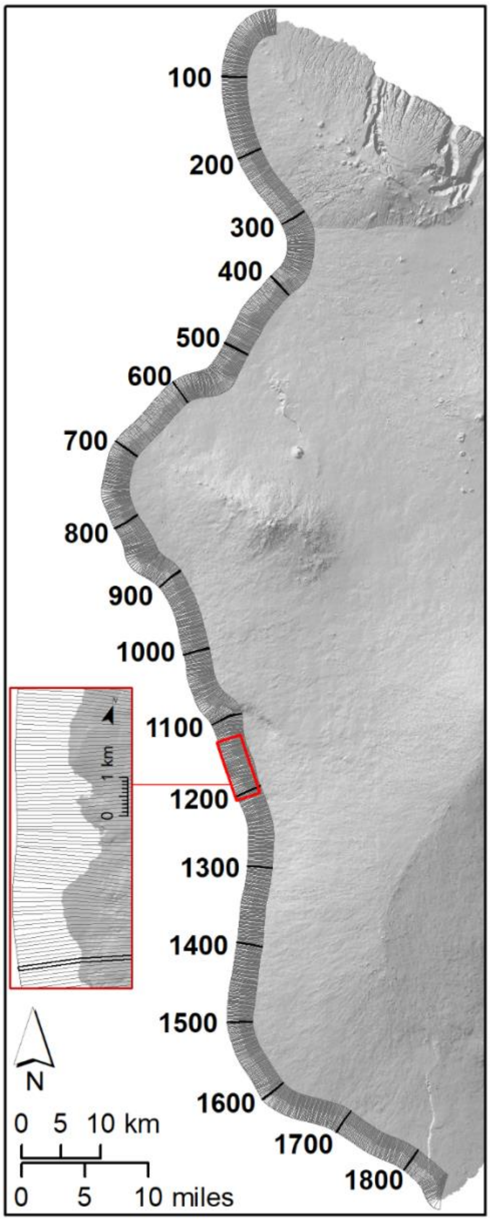

Fig. S29. The 100-m width coastal sectors along our study area numbered ascending from north to south (labelled every 100 sectors, or approximately every 10 km), with inset map showing detailed view of sectors.

References

1 Maynard, J. *et al.* Relative resilience potential and bleaching severity in the West Hawai'i Habitat Focus Area in 2015. *NOAA Technical Memorandum*, 53 pp (2016).

2 Beijbom, O. *et al.* Towards Automated Annotation of Benthic Survey Images: Variability of Human Experts and Operational Modes of Automation. *PLoS ONE* **10**, e0130312 (2015).

3 Wedding, L. M. *et al.* Advancing the integration of spatial data to map human and natural drivers on coral reefs. *PLoS ONE* **13** (2018).

4 Graham, N. A. J. *et al.* Changing role of coral reef marine reserves in a warming climate. *Nat. Comm.* **11**, 2000 (2020).

5 Anderson, M. J., Walsh, D. C. I., Robert Clarke, K., Gorley, R. N. & Guerra-Castro, E. Some solutions to the multivariate Behrens–Fisher problem for dissimilarity-based analyses. *Aust. N. Z. J. Stat.* **59**, 57-79 (2017).

6 Anderson, M., Gorley, R. N. & Clarke, R. K. *Permanova+ for primer: Guide to software and statisticl methods*. (Primer-E Limited, 2008).

7 Clarke, K. & Gorley, R. Getting started with PRIMER v7. *PRIMER-E: Plymouth, Plymouth Marine Laboratory* **20** (2015).

8 Anderson, M. J. & Willis, T. J. Canonical Analysis of Principal Coordinates: A Useful Method of Constrained Ordination for Ecology. *Ecology* **84**, 511-525 (2003).

9 Côté, I. M., Gill, J. A., Gardner, T. A. & Watkinson, A. R. Measuring coral reef decline through meta-analyses. *Philos. Trans. R. Soc. Ser. B: Biol. Sci.* **360**, 385-395 (2005).

10 Graham, N. A. J. *et al.* Climate Warming, Marine Protected Areas and the Ocean-Scale Integrity of Coral Reef Ecosystems. *PLoS ONE* **3**, e3039 (2008).

11 Smith, J. E. *et al.* Re-evaluating the health of coral reef communities: baselines and evidence for human impacts across the central Pacific. *Proc. R. Soc. B.* **283**, 20151985 (2016).

12 Donovan, M. K. *et al.* Combining fish and benthic communities into multiple regimes reveals complex reef dynamics. *Sci. Rep.* **8**, 16943 (2018).

13 Jouffray, J.-B. *et al.* Identifying multiple coral reef regimes and their drivers across the Hawaiian archipelago. *Philos. Trans. R. Soc. Ser. B: Biol. Sci.* **370**, 20130268 (2015).

14 Williams, G. J., Gove, J. M., Eynaud, Y., Zgliczynski, B. J. & Sandin, S. A. Local human impacts decouple natural biophysical relationships on Pacific coral reefs. *Ecography* **38**, 751-761 (2015).

15 MuMln: Multi-Model Inference. R package version 1.13.4. (2015).

16 Hurvich, C. M. & Tsai, C.-L. Regression and time series model selection in small samples. *Biometrika* **76**, 297-307 (1989).

17 Asner, G. P. *et al.* Mapped coral mortality and refugia in an archipelago-scale marine heat wave. *Proc. Natl. Acad. Sci.* **119**, e2123331119 (2022).

18 Brant, R. Assessing Proportionality in the Proportional Odds Model for Ordinal Logistic Regression. *Biometrics* **46**, 1171-1178 (1990).

19 McFadden, D. Conditional logit analysis of qualitative choice behavior. *Academic Press New York* (1974).

20 Burnham, K. P. Model selection and multimodel inference. *A practical information-theoretic approach* (1998).

21 Richards, S. A. Testing ecological theory using the information-theoretical approach: examples and cautionary results *Ecology* **86**, 2805-2814 (2005).
22 Battista, T., Costa, B. & Anderson, S. Atlas of the shallow-water benthic habitats of the main Hawaiian Islands. *NOAA Technical Memorandum NOS NCCOS* **61**, 205-223 (2007).
23 Whittier, R. B. & El-Kadi, A. I. Human health and environmental risk of onsite sewage disposal systems for the Hawaiian Islands of Kauai, Maui, Molokai, and Hawaii *Hawaii Dept. of Health* (2014).
24 DOH. Underground Injection Control Permit application files. *Hawaii Dept. of Health* (2017).
25 DOH. Individual Wastewater System Database. *Hawaii Dept. of Health* (2017).
26 Delevaux, J. M. S. *et al.* A linked land-sea modeling framework to inform ridge-to-reef management in high oceanic islands. *PLoS ONE* **13**, e0193230 (2018).
27 Brosnan JT & J, D. Bermudagrass. *Turf Management* (2008).
28 GCSAA. Nutrient Use and Management Practices on U.S. Golf Courses. *Golf Course Environmental Profile* **II** (2016).
29 Kunimatsu, T., Sudo, M. & Kawachi, T. Loading rates of nutrients discharging from a golf course and a neighboring forested basin. *Water Sci. Technol.* **39**, 99-107 (1999).
30 Shuman, L. M. Phosphate and Nitrate Movement Through Simulated Golf Greens. *Water, Air, Soil Pollut.* **129**, 305-318 (2001).
31 Wong, J. W. C., Chan, C. W. Y. & Cheung, K. C. Nitrogen and Phosphorus Leaching from Fertilizer Applied on Golf Course: Lysimeter Study. *Water, Air, Soil Pollut.* **107**, 335-345 (1998).
32 Rea, A. & Skinner, K. D. Geospatial datasets for watershed delineation and characterization used in the Hawai ʻi StreamStats web application. *US Geol. Surv. Data Ser.* **680**, 12 (2012).
33 Longman, R. J. *et al.* Compilation of climate data from heterogeneous networks across the Hawaiian Islands. *Sci. Data* **5**, 180012 (2018).
34 Longman, R. J., Newman, A. J., Giambelluca, T. W. & Lucas, M. Characterizing the Uncertainty and Assessing the Value of Gap-Filled Daily Rainfall Data in Hawaii. *J. Appl. Met. Clim.* **59**, 1261-1276 (2020).
35 Sharp, R. *et al.* InVEST Version 3.2. 0 User's Guide. The Natural Capital Project. The Nature Conservancy, and World Wildlife Fund. (2015).
36 Falinski, K. A. *Predicting sediment export into tropical coastal ecosystems to support ridge to reef management*, University of Hawai'i at Manoa, (2016).
37 Hamel, P., Chaplin-Kramer, R., Sim, S. & Mueller, C. A new approach to modeling the sediment retention service (InVEST 3.0): Case study of the Cape Fear catchment, North Carolina, USA. *Sci. Total Environ.* **524-525**, 166-177 (2015).
38 NRCS. Estimation of Direct Runoff from Storm Rainfall; National Engineering Handbook–Part 630 Hydrology, Chapter 10:. *U.S. Dept. Agr.: Nat. Res. Conserv. Serv.* (2004).
39 Service, N. R. C. in *National Engineering Handbook, Part 630 Hydrology* Ch. 9, (2004).
40 Glysson, G. D. Sediment-transport curves. Report No. 87-218, (1987).
41 Skirving, W. *et al.* CoralTemp and the Coral Reef Watch Coral Bleaching Heat Stress Product Suite Version 3.1. *Remote Sens.* **12**, 3856 (2020).

42 Gove, J. M. *et al.* Quantifying Climatological Ranges and Anomalies for Pacific Coral Reef Ecosystems. *PLoS ONE* **8**, e61974 (2013).

43 Stopa, J. E. *et al.* Wave energy resources along the Hawaiian Island chain. *Renew. Energy* **55**, 305-321 (2013).

44 Li, N. *et al.* Thirty-four years of Hawaii wave hindcast from downscaling of climate forecast system reanalysis. *Ocean Model. Online* **100**, 78-95 (2016).

45 Tolman, H. L. A mosaic approach to wind wave modeling. *Ocean Model. Online* **25**, 35-47 (2008).

46 Booij, N., Ris, R. C. & Holthuijsen, L. H. A third-generation wave model for coastal regions: 1. Model description and validation. *J. Geophys. Res. (Oceans)* **104**, 7649-7666 (1999).

47 Saha, S. *et al.* The NCEP Climate Forecast System Reanalysis. *Bull. Am. Met. Soc.* **91**, 1015-1058 (2010).

48 Saha, S. *et al.* The NCEP Climate Forecast System Version 2. *J. Clim.* **27**, 2185-2208 (2014).

49 Skamarock, W. C. *et al.* A description of the advanced research WRF version 2. *National Center For Atmospheric Research* (2005).

50 Powers, J. G. *et al.* The Weather Research and Forecasting Model: Overview, System Efforts, and Future Directions. *Bull. Am. Met. Soc.* **98**, 1717-1737 (2017).

51 Gove, J. M. *et al.* Coral reef benthic regimes exhibit non-linear threshold responses to natural physical drivers. *Mar. Ecol. Prog. Ser.* **522**, 33-48 (2015).
