# Supplementary material for: Coral reefs benefit from reduced land–sea impacts under ocean warming
Source: Nature. 2023 Aug 9;621(7979):536–42. doi: 10.1038/s41586-023-06394-w (PMC10511326; doi:10.1038/s41586-023-06394-w)
Supplement: Supplementary file 2 — Reporting Summary [file 41586_2023_6394_MOESM2_ESM.pdf]

nature portfolio

Corresponding author(s): Jamison Gove, Gareth Williams

Last updated by author(s): Aug 7, 2023

Reporting Summary

Nature Portfolio wishes to improve the reproducibility of the work that we publish. This form provides structure for consistency and transparency in reporting. For further information on Nature Portfolio policies, see our Editorial Policies and the Editorial Policy Checklist.

Statistics

For all statistical analyses, confirm that the following items are present in the figure legend, table legend, main text, or Methods section.

n/a	Confirmed	
☐	☒	The exact sample size (*n*) for each experimental group/condition, given as a discrete number and unit of measurement
☐	☒	A statement on whether measurements were taken from distinct samples or whether the same sample was measured repeatedly
☐	☒	The statistical test(s) used AND whether they are one- or two-sided<br>*Only common tests should be described solely by name; describe more complex techniques in the Methods section.*
☐	☒	A description of all covariates tested
☐	☒	A description of any assumptions or corrections, such as tests of normality and adjustment for multiple comparisons
☐	☒	A full description of the statistical parameters including central tendency (e.g. means) or other basic estimates (e.g. regression coefficient) AND variation (e.g. standard deviation) or associated estimates of uncertainty (e.g. confidence intervals)
☐	☒	For null hypothesis testing, the test statistic (e.g. *F*, *t*, *r*) with confidence intervals, effect sizes, degrees of freedom and *P* value noted<br>*Give P values as exact values whenever suitable.*
☒	☐	For Bayesian analysis, information on the choice of priors and Markov chain Monte Carlo settings
☐	☒	For hierarchical and complex designs, identification of the appropriate level for tests and full reporting of outcomes
☐	☒	Estimates of effect sizes (e.g. Cohen's *d*, Pearson's *r*), indicating how they were calculated

Our web collection on statistics for biologists contains articles on many of the points above.

Software and code

Policy information about availability of computer code

Data collection: All data and code that support the findings of this study are available at https://github.com/jamisongove/Coral-Reef-Persistence.

Data analysis: Statistical analyses were performed using the software packages R (www.r-project.org) version 4.0.2 (using libraries gamm4, MuMIn, foreach, doMC, ggplot2, gmt, tidyverse, zoo, lubridate) (ref. 1), Matlab (www.matthworks.com) using v2021a (using Statistics and Machine Learning toolbox), ArcGIS Desktop (www.esri.com) v10.6 with Advanced licensing and extensions Spatial Analyst and Geostatistical Analyst, InVEST Sediment Delivery Ratio model (https://naturalcapitalproject.stanford.edu/software/invest), and the PERMANOVA+ (ref. 2) add-on for Primer version 7 (ref. 3).

1 Team, R. C. in R Roundation for Statistical Computing (2021).
2 Anderson, M., Gorley, R. N. & Clarke, R. K. Permanova+ for primer: Guide to software and statistical methods (2008).
3 Clarke, K. & Gorley, R. Getting started with PRIMER v7. PRIMER-E: Plymouth, Plymouth Marine Laboratory (2015).

For manuscripts utilizing custom algorithms or software that are central to the research but not yet described in published literature, software must be made available to editors and reviewers. We strongly encourage code deposition in a community repository (e.g. GitHub). See the Nature Portfolio guidelines for submitting code & software for further information.

Data

Policy information about availability of data

All manuscripts must include a data availability statement. This statement should provide the following information, where applicable:

- Accession codes, unique identifiers, or web links for publicly available datasets
- A description of any restrictions on data availability
- For clinical datasets or third party data, please ensure that the statement adheres to our policy

All data that support the findings of this study are available at https://github.com/jamisongove/Coral-Reef-Persistence. Reef fish length-weight parameters were obtained from FishBase (https://fishbase.org) and ref. 1, human population data from NASA Gridded Population of the World v4 (https://sedac.ciesin.columbia.edu/data/set/gpw-v4-population-count-rev11), land use and land cover data from the NOAA Coastal Change Analysis Program (https://www.coast.noaa.gov/htdata/raster1/landcover/bulkdownload/), soils data from USDA Gridded Soil Survey Geographic Database (gSSURGO; https://www.nrcs.usda.gov/resources/data-and-reports/gridded-soil-survey-geographic-gssurgo-database), sub-watershed catchment data from USGS Stream Stats (https://water.usgs.gov/GIS/metadata/usgswrd/XML/ds680_archydrohucs.xml) (ref. 2), watershed and digital elevation model data from USGS National Hydrography Dataset (https://www.usgs.gov/national-hydrography/national-hydrography-dataset), rainfall data from refs. 3,4, Landsat 8 satellite image from USGS (https://earthexplorer.usgs.gov/), Landsat 7 and 8 cloud-free composites derived using Google Earth Engine (https://earthengine.google.com/), individual wastewater systems for Hawaiʻi from refs. 5,6, marine managed area designation from ref. 80 and downloadable from the State of Hawaiʻi (https://planning.hawaii.gov/gis), fishing regulations from the State of Hawaiʻi (https://dlnr.hawaii.gov/dar/fishing/fishing-regulations/), sea surface temperature and degree heating week data from NOAA Coral Reef Watch (https://coralreefwatch.noaa.gov/product/5km), ocean color (chlorophyll-a and irradiance) data from NOAA Coral Reef Watch (https://coralreefwatch.noaa.gov/product/oc/index.php) and ref. 7. See Methods and Supplemental Information for more detailed information on the data used to support the findings of this study.

1 Donovan, M. K. et al. Combining fish and benthic communities into multiple regimes reveals complex reef dynamics. Sci. Rep. 8, 16943 (2018).
2 Rea, A. & Skinner, K. D. Geospatial datasets for watershed delineation and characterization used in the Hawaiʻi StreamStats web application. US Geol. Surv. Data Ser. 680, 12 (2012).
3 Longman, R. J., Newman, A. J., Giambelluca, T. W. & Lucas, M. Characterizing the Uncertainty and Assessing the Value of Gap-Filled Daily Rainfall Data in Hawaii. J. Appl. Met. Clim. 59, 1261-1276 (2020).
4 Longman, R. J. et al. Compilation of climate data from heterogeneous networks across the Hawaiian Islands. Scientific Data 5, 180012 (2018).
5 DOH. Individual Wastewater System Database. Hawaii Dept. of Health (2017).
6 DOH. Underground Injection Control Permit application files. Hawaii Dept. of Health (2017).
7 Wedding, L. M. et al. Advancing the integration of spatial data to map human and natural drivers on coral reefs. PLoS ONE 13 (2018).

Human research participants

Policy information about studies involving human research participants and Sex and Gender in Research.

Reporting on sex and gender	NOT APPLICABLE
Population characteristics	NOT APPLICABLE
Recruitment	NOT APPLICABLE
Ethics oversight	NOT APPLICABLE

Note that full information on the approval of the study protocol must also be provided in the manuscript.

Field-specific reporting

Please select the one below that is the best fit for your research. If you are not sure, read the appropriate sections before making your selection.

☐ Life sciences ☐ Behavioural & social sciences ☒ Ecological, evolutionary & environmental sciences

For a reference copy of the document with all sections, see nature.com/documents/nr-reporting-summary-flat.pdf

Ecological, evolutionary & environmental sciences study design

All studies must disclose on these points even when the disclosure is negative.

Study description	The study tested the hypothesis that mitigating local human impacts facilitates coral reef persistence in the face of climate change-induced disturbance, specifically mass coral bleaching. Our goal was to move beyond commonly used proxies of local human impacts and generate spatially resolved data on specific land-sea human activities to identify actionable outcomes. This further allowed us to quantify the effects mitigating either land- or sea-based human impacts in isolation or simultaneously had on the ability of key reef-building organisms to recover post-disturbance. We achieved this by combining recurring in-water SCUBA surveys of coral reef benthic and fish communities with a 20-year time series of land-sea human impacts and other environmental factors thought to drive

coral reef ecosystem processes. Our study included reefs across a broad range of ecological states, large spatiotemporal gradients in land-sea human impacts and environmental factors, and which experienced the most severe marine heatwave on record in the Hawaiian Islands.

Research sample	We quantified changes in the per cent cover of major reef-building benthic groups (hard coral, crustose coralline algae) and related these to concurrent changes in numerous land-sea human impacts, including urban runoff, wastewater pollution, nutrient loading, sediment input, and local restrictions on fishing gear types. Environmental factors included peak and annual rainfall, wave exposure, variability in ocean temperatures and heat stress, irradiance, and phytoplankton biomass. We also incorporated multiple fish biomass metrics that represent the critical role reef fish play in maintaining coral reef ecosystem dynamics. All human impacts and environmental factors were chosen based on prior evidence in the literature that they represent key drivers of reef ecosystem processes and were quantified using a variety of modelled and satellite-derived data sources.
Sampling strategy	Underwater visual surveys of shallow-water benthic and reef-fish assemblages were collated from the following three coral reef ecosystem monitoring agencies to maximise spatial and temporal replication across the study region: State of Hawaiʻi Division of Aquatic Resources, National Park Service, and The Nature Conservancy. Each program conducted surveys using similar data collection methods (see below) in shallow-water (<30 m) depths over hard-bottom substrate.
Data collection	All coral reef surveys used a traditional 25 m belt-transect method. Benthic surveys used permanently marked pins to ensure the same area of reef was surveyed over time. High resolution photographs were collected via photoquadrats at 1 m intervals along 25 m belt-transects (N = 26 photographs per transect). Thirty to fifty random points were overlaid on each photograph and the benthic component under each point was identified to the lowest possible taxonomic level. Per cent cover of the major functional groups were used in this analysis, namely hard coral, crustose coralline algae, macroalgae, and turf algae. All data were averaged among each transect and then among all transects for each site (1 – 4 transects per site, per year, depending on the monitoring program). Surveys of reef-fish assemblages were performed along the same permanently marked 25 m transects concurrently with benthic surveys. In all surveys, fishes were identified to species, sized, and enumerated. To account for differences among programs in how researchers surveyed reef fish, counts were calibrated using species and method specific adjustments previously developed for the region.
Timing and spatial scale	Underwater visual surveys of benthic assemblages were collated from three monitoring programs for the following years (number of reefs surveyed are in parentheses): 2003 (23), 2007 (23), 2011 (23), 2014 (40), 2015 (40), 2016 (80), 2017 (80), 2018 (15), 2019 (55). All benthic surveys used permanently marked pins to ensure the same area of reef was surveyed over time. High resolution photographs were collected via photoquadrats at 1 m intervals along 25 m belt-transects (N = 26 photographs per transect). Thirty to fifty random points were overlaid on each photograph and the benthic component under each point was identified to the lowest possible taxonomic level. Per cent cover of the major functional groups at each reef were used in this analysis, namely hard coral and crustose coralline algae. Surveys of reef-fish assemblages were performed along the same permanently marked 25 m transects concurrently with benthic surveys. However, reef fish surveys were performed more frequently (1 – 6 times per year from 2003 – 2019) than benthic surveys, depending on the reef location and monitoring program performing the surveys. In all surveys, fishes were identified to species, sized, and enumerated. To account for differences among programs in how researchers surveyed reef fish, counts were calibrated using species and method specific adjustments. The survey region spanned roughly 200 km of coastline on the island of Hawaiʻi.
Data exclusions	Fish species were excluded from fish biomass calculations according to life history characteristics that are not well captured with visual surveys, including cryptic benthic species, nocturnal species, pelagic schooling species, and manta rays. We also accounted for extreme observations of schooling species, which were defined by calculating the upper 99.9% of all individual observations, resulting in 26 observations out of over 0.5 million, comprised of 11 species. The distribution of individual counts in the entire database for those 11 species was then used to identify observations that fell above the 99.0% quantile of counts for each species individually. These observations were adjusted to the 99.0% quantile for analysis.<br><br>Other data exclusions include outliers in predictor variables (the local human impacts and environmental factors). Within the section "Coral reef trajectories pre-disturbance", prior to calculating per cent difference, we identified and removed outliers that fell outside a threshold of ± 2 standard deviations of the median. Within the section "Coral response to the 2015 Marine Heatwave", prior to model fitting, we identified the presence of outliers in our predictor variables as any point that fell outside a threshold of ± 2 standard deviations of the median. We then applied an additional step to retain any point above this threshold that was within 25% of the maximum predictor value below the threshold. This ensured that no data points were unnecessarily discarded from our formal model-fitting process because of applying an arbitrary threshold cutoff for data inclusion. We used the exact same process to identify and remove outliers within the section "Coral reefs four years post-disturbance" prior to formal model fitting.
Reproducibility	A description of the methodologies used is provided in the Methods and expanded on substantially for several of the human impact and environmental factors in the Supplementary Information. The data and full code necessary to reproduce the findings are available at https://github.com/jamisongove/Coral-Reef-Persistence
Randomization	Survey sites were either randomly or haphazardly chosen by the various monitoring agencies involved in data collection. Sites were separated by a minimum distance (250 m) and transects within sites were also separated by a minimum distance (5 - 10 m). To minimise observer bias of fish counts, sizing calibration dives were conducted using fish models of known size at the beginning of each field season. Observer crossover training was done using two observers side by side when possible. Benthic cover estimates were quantified by randomly assigning 20 points to each image using post-hoc image analysis programs (Photogrid or Coral Point Count with Excel Extensions) and identifying the benthic group to the lowest taxonomic rank under each point.
Blinding	All in situ benthic and reef fish surveys were conducted prior to this research question being conceived. The divers carried out the surveys for the most part without prior knowledge of the local human impacts and environmental factors for their respective survey locations – we later quantified these for each reef location and time of survey, thus blinding in this respect was achieved. In some cases, divers were aware of any local fishing restrictions in effect, but this was unavoidable as many of them specifically survey inside and outside of these zones

Did the study involve field work? ☒ Yes ☐ No

Field work, collection and transport

Field conditions	Because of the nature of collecting underwater benthic information, field conditions must be relatively calm (i.e., low wind and wave activity) with relatively good underwater visibility (i.e., > 5 m).
Location	Our study site was Hawaiʻi Island (19.55°N, 155.66°W), USA, which is the southeastern most island of the Hawaiian Archipelago, located in the northern central Pacific. The western section has roughly 200 km of coastline that is predominately oriented north to south. The coastline contains the longest contiguous reef ecosystem in the main Hawaiian Islands and large gradients in human population, local land-sea impacts, and environmental factors. The region represents an ideal study location for resolving the interacting land-sea human impacts driving reef ecosystem change and coral trajectories following acute climate-driven disturbance. All reefs included in this study were in shallow-water (depth < 30 m).
Access & import/export	All survey data were collected with the knowledge and consent of the State of Hawaiʻi, which has legal jurisdiction of all waters from 0 – 3 nm of the shoreline. The director of the State of Hawaiʻi's Division of Aquatic Resources, which is the managing agency of State waters, contributed survey data and both a collaborator and coauthor on this manuscript.
Disturbance	All surveys were performed by professional scientific divers that aim to minimise contact and disturbance of the reef. No coral reef benthic or fish species were removed from their habitat as part of this effort

Reporting for specific materials, systems and methods

We require information from authors about some types of materials, experimental systems and methods used in many studies. Here, indicate whether each material, system or method listed is relevant to your study. If you are not sure if a list item applies to your research, read the appropriate section before selecting a response.

Materials & experimental systems

n/a	Involved in the study	
☒	☐	Antibodies
☒	☐	Eukaryotic cell lines
☒	☐	Palaeontology and archaeology
☒	☐	Animals and other organisms
☒	☐	Clinical data
☒	☐	Dual use research of concern

Methods

n/a	Involved in the study	
☒	☐	ChIP-seq
☒	☐	Flow cytometry
☒	☐	MRI-based neuroimaging
